# Curcumin coacervates for supramolecular-interaction-responsive cytosolic siRNA delivery to enhance pyroptosis

**DOI:** 10.7150/thno.121865

**Published:** 2026-01-01

**Authors:** Kai Cheng, Fang Zhang, Yishu Bao, Zhiyi Xu, Hao Kong, Dingdong Yuan, Zhong Zheng, Yuan-Di Zhao, Jiang Xia

**Affiliations:** 1 Department of Chemistry, The Chinese University of Hong Kong, Shatin, Hong Kong SAR, P. R. China.; 2 Britton Chance Center for Biomedical Photonics at Wuhan National Laboratory for Optoelectronics-Hubei Bioinformatics & Molecular Imaging Key Laboratory, Department of Biomedical Engineering, College of Life Science and Technology, Huazhong University of Science and Technology, Wuhan 430074, Hubei, P. R. China.

**Keywords:** curcumin coacervates, supramolecular interaction, cyclodextrin-responsive release, cytosolic delivery, enhanced pyroptosis

## Abstract

**Rationale:** Synthetic molecules, meticulously designed according to the “sticker-and-spacer model”, tend to form coacervates via liquid-liquid phase separation (LLPS), thereby acquiring properties beyond their discrete and soluble states. However, natural compounds, such as those from traditional Chinese medicines (TCMs), are not known to undergo phase separation. In this study, we demonstrate that curcumin, the active ingredient in the spice turmeric, forms phase-separated fluorescent coacervates when diluted from a concentrated organic-solvent solution into an aqueous solution.

**Methods:** Curcumin coacervates were formed by diluting a concentrated stock solution in organic solvents into the aqueous solution. We utilized the coacervate droplets to encapsulate and transport various biomacromolecules, such as proteins and nucleic acids, across the plasma membrane into the cell. Supramolecular interaction between β-cyclodextrin (β-CD) and curcumin disassembles curcumin coacervates, leading to cargo release in the cytosol.

**Results:** Intravenously injected curcumin coacervates spontaneously enrich in the tumor tissue in tumor-bearing BALB/c mice. Subsequent intratumoral injection of β-CD significantly enhances anticancer effects in mice, demonstrating the efficacy of coacervate-mediated siRNA drug delivery and supramolecular-interaction-responsive intracellular release *in vivo*.

**Conclusions:** Taken together, we report here the coacervate-forming properties of the natural TCM compound curcumin, presenting a unique strategy for controlling coacervate states through supramolecular interactions with β-cyclodextrin *in vitro* and *in vivo*, along with the unexplored potential of curcumin coacervate-mediated siRNA delivery to enhance pyroptosis.

## Introduction

Liquid-liquid phase separation (LLPS) describes a physical phenomenon where molecules form highly concentrated liquid condensates in an aqueous solution, driven by weak interactions between molecules such as hydrogen bonds, electrostatic forces, and hydrophobic interactions, *etc*. 1-4. These condensates are separated from the bulk solution by a well-defined boundary, across which concentrations of the two phases differ by several orders of magnitude. Yet, molecules in the condensed phase and the dilute phase dynamically exchange. Initially recognized as a common occurrence in polymer solutions, LLPS of biopolymers has recently been observed in cells, which form membraneless organelles like nucleoli and stress bodies, compartmentalizing cellular space and regulating various cellular processes, including gene transcription, heterochromatin formation, spindle apparatus assembly, asymmetric cell division, autophagy, innate immune response, and many others 5-10. In addition to contributing to regular cellular physiology, LLPS is also linked to the emergence and progression of diseases. For instance, in neurodegenerative diseases, improper LLPS may lead to protein aggregation, triggering the disease process 11, 12. Recently, studies have found that small molecules may also undergo spontaneous phase separation. For instance, molecules whose structures meet the “sticker-and-spacer” model tend to form droplets in aqueous solutions 13, 14. For example, our group designed a compound with two pyrene moieties separated by a polyethylene glycol linker and discovered that it formed fluorescent coacervates in solution 15. Despite covering a broad spectrum of chemical space, natural products have yet to be reported as exhibiting phase separation behavior.

Curcumin is a traditional natural medicine compound extracted from the rhizome of *Curcuma longa* and has gained attention due to its wide range of biological activities and potential therapeutic effects. Curcumin has been shown to reduce inflammatory responses by inhibiting nuclear factor κB (NF-κB), pro-inflammatory cytokines (such as TNF-α and IL-6), and other inflammatory signaling pathways 16-19. It has also been demonstrated to treat diseases related to chronic inflammation, such as rheumatoid arthritis and inflammatory bowel disease 20-22. Moreover, curcumin is known to scavenge free radicals, inhibit lipid peroxidation, and enhance the activity of intracellular antioxidant enzymes, including superoxide dismutase and glutathione peroxidase 23-25. In addition to its anti-inflammatory properties, curcumin exhibits anticancer activities such as inducing cell death, inhibiting tumor cell proliferation, and suppressing tumor angiogenesis 26-29. Furthermore, curcumin is widely used as a colorant in the dyeing industry 30, 31. Although curcumin is known for its low solubility in solution, it remains unclear whether curcumin can exist in other physical states, such as phase-separated coacervates.

On another note, the coacervate state endows small molecules with new properties that are unattainable in other states. One such property is that molecular coacervates tend to encapsulate biological molecules, such as proteins and siRNA. The second property is that coacervates often interact with biological membranes and enter membrane-bound vesicles such as liposomes or cells 15, 32, 33. Therefore, coacervates formed by small molecules may act as vehicles to passively transport biological molecules into cells. However, the cargo may still be trapped within the droplets inside cells and cannot be easily released into the cytosol to perform their biological functions. To address this limitation, researchers have developed various mechanisms that use external stimuli to dissipate the coacervates and release the encapsulated cargo into the cytoplasm. For instance, a phase-separating peptide HBpep-SR can release protein cargo by reacting with intracellular glutathione: in the cytosol, glutathione-mediated reduction removes the side chain group on lysine, disassembling the phase-separated coacervates and releasing the payload 34, 35. Light is also an effective stimulus. We designed low-molecular-weight small molecules that can form coacervates, transport proteins into cells, and utilize chemical reactions to regulate cargo distribution within the cell. We designed a photo-responsive, phase-separating fluorescent molecule (PPFM) with a molecular weight of 666.6 Daltons based on pyrene. The PPFM undergoes LLPS in the aqueous solution and forms coacervates that carry payloads into cells, respond to a benign stimulus - 405 nm light, and release the protein cargo to the cytosol 15. Recently, we designed another group of small molecules consisting of two triarylphosphine groups connected by a polyethylene glycol linker. Ethyl azidoacetate reacts with the triarylphosphine to dissolve the coacervates and release the cargo into the cytosol 36. We also reported a peptide with Fmoc at one end and a DOPA group at the other end that formed coacervates 37.

**Scheme 1:**
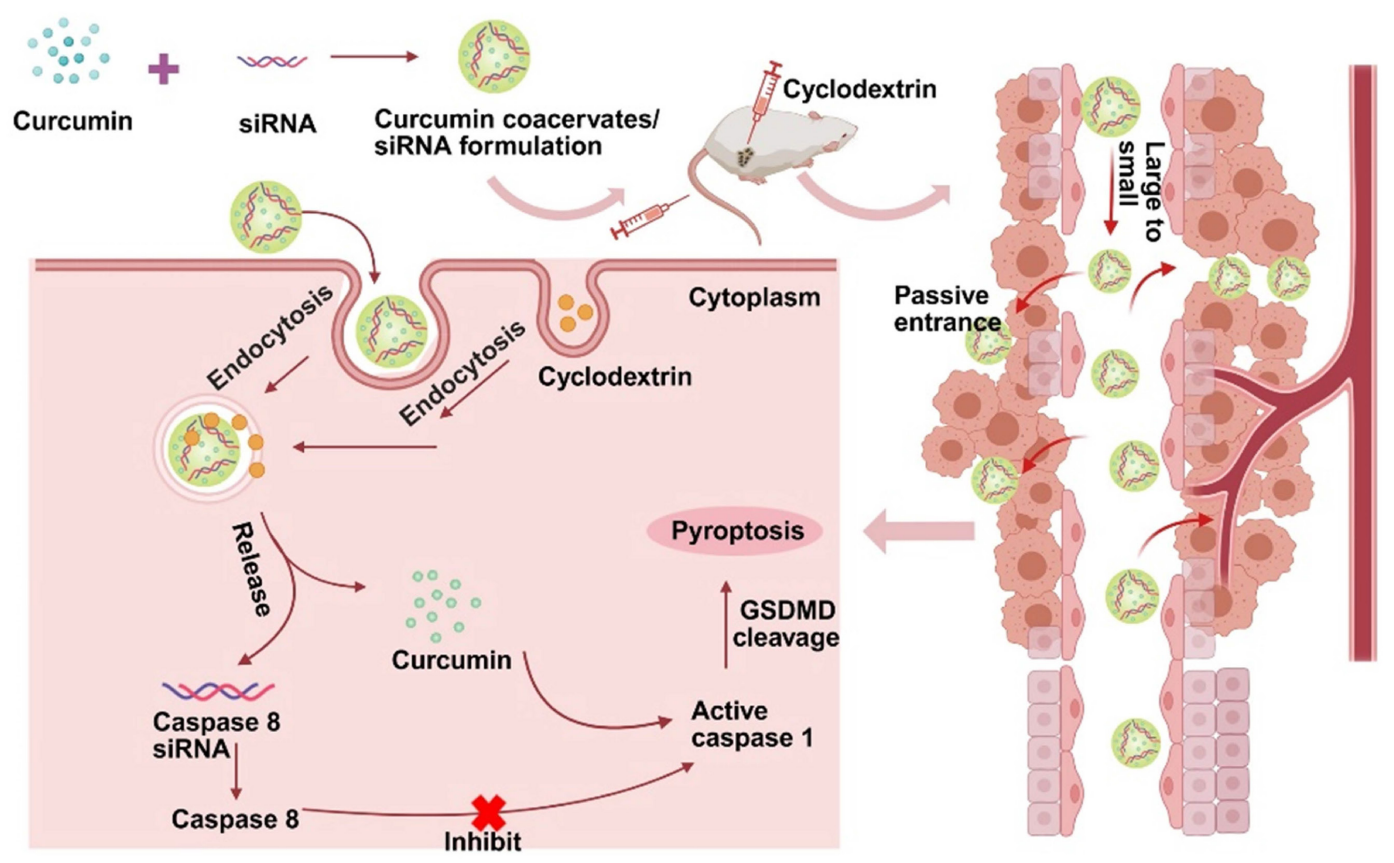
** Schematic diagram of the anti-tumor mechanism of the caspase 8 siRNA via curcumin coacervate-mediated delivery and β-CD-induced release.** Briefly, when curcumin coacervates loaded with caspase 8 siRNA were injected into CT26 tumor-bearing BALB/c mice through the tail vein, they were enriched in the tumor, likely through the enhanced permeability and retention effect, and subsequently entered the tumor cells. After intratumoral injection of β-CD, curcumin and siRNA were released from coacervates. Curcumin can activate caspase 1, induce cell membrane perforation, and cell rupture. Caspase 8 siRNA reduces the synthesis of caspase 8, an inhibitor of caspase 1, which also leads to activation of caspase 1, thereby further achieving cell pyroptosis and inhibiting tumor growth. Scheme 1 was created in BioRender. Cheng, K. (2025) https://BioRender.com/9y6n661.

In this work, we demonstrate that curcumin forms fluorescent, phase-separated coacervates under certain conditions. Curcumin coacervates can act as delivery vehicles to translocate proteins, nucleic acids, and small molecules into cells. β-cyclodextrin (β-CD) disassembles the coacervates within the cell and releases the cargo into the cytosol. We present one application of curcumin coacervates in cancer treatment: curcumin coacervates deliver siRNA into cancer cells, and β-CD induces cytosolic release of siRNA, which exhibits pronounced anticancer effects by enhanced pyroptosis *in vivo* (**Scheme 1**).

## Materials and Methods

### Materials and instruments

All reagents and solvents were purchased without further purification. Curcumin was purchased from Sigma-Aldrich; Roswell Park Memorial Institute (RPMI) 1640, fetal bovine serum (FBS), and Penicillin-Streptomycin-Glutamine (PS, 100×) were purchased from Thermo Fisher Scientific Inc; cell counting kit-8 was purchased from Med Chem Express; Lyso-Tracker Red, Golgi-Tracker Red, ER- Tracker Red, Caspase-1 and -4 activity analysis kit, cell cycle analysis kit, and hoechst33342 were purchased from Shanghai Beyotime Biotechnology Co., Ltd.; 5-week-old BALB/c mice (SPF grade) were purchased from Hunan Silaikejingda Experimental Animal Co., Ltd. Mice were housed in an animal facility under constant environmental conditions (room temperature, 22 ± 1°C; relative humidity, 40-70%; and a 12 h light-dark cycle), and all mice had access to food and water ad libitum. All animal experiments were approved by the Animal Experiment Ethics Committee of Huazhong University of Science and Technology (IACUC number: 4207).

### Preparation and characterization of curcumin coacervates

Curcumin was dissolved in DMSO (100 mg/mL, stock solution), and 10 μL of stock solution was dispersed in ultrapure water to form a turbid solution. Then, the two- and three-dimensional morphologies and the fusion of coacervate droplets over time were observed under a confocal microscope. Fluorescence recovery after photobleaching (FRAP) (laser: 488 nm, intensity: 25%, time: 5 s) was performed. Coacervate droplets of different concentrations (25, 12.5, 6.25, 3.12, 1.56, 0.78, 0.39, 0.2, 0.1, 0.05, 0.025, 0.01, 0.005, 0.0025, and 0.001 mg/mL) were prepared to measure the turbidity absorption at 600 nm. The morphology of 25, 3.12, 0.01, and 0.005 mg/mL curcumin droplets was observed under a confocal microscope.

Phase diagrams of curcumin coacervate droplets were prepared at different concentrations (0.0015, 0.003, 0.006, 0.012, 0.025, 0.005, and 0.1 mg/mL) and pH (1, 3, 5, 7, 9, 11, and 13), and the droplet morphology was observed under some concentrations and pH conditions. In addition, 5 mg/mL coacervate droplets were prepared at different NaCl concentrations (1, 10, 100, and 1000 mM), and turbidity was measured, and the morphology was observed.

Curcumin coacervate droplets (10 mg/mL) were respectively mixed with small molecule (Nlie Red, RhB, Rh6G, PTSA, Cy3) and macromolecule (Cy5-IgG, Cy5-BSA, Cy5-β-gal, Cy5-lysozyme, and Cy5-ssDNA), all the molecule concentration were 0.1 mg/mL, then centrifuged at 1000 r for 3 min, the supernatant was taken to detect the characteristic absorption peak, and the recruitment efficiency of the small molecule was calculated. The precipitate was re-dissolved in ultrapure water, and the morphology was observed under a confocal microscope.

### β-CD-induced release of curcumin coacervate droplets

For drawing a standard curve, curcumin molecular solutions (dissolved in DMSO) of different concentrations were prepared to measure the characteristic absorption peak at 430 nm. Five equal portions of 2 mg of curcumin powder were added to solutions of β-CD at different concentrations (0.16, 0.32, 0.48, 0.64, and 0.8 mg/mL). After stirring thoroughly for 0.5 h, the solution was centrifuged (2000 rpm, 5 min) to remove the precipitate, and the supernatant was then measured for absorbance at 430 nm. For infrared spectrum analysis, 2 mL of β-CD saturated solution (31.8 mg/mL) was prepared, and 1mg of curcumin powder was added with full stirring for 0.5 h, then the solution was filtered to collect the precipitate, and dried. To study the effect of different concentrations of β-CD on the dissolution and release of curcumin, 1 mg/mL curcumin coacervate droplets were prepared. Different concentrations of β-CD (0.3, 0.6, 1.2, 2.5, 5, and 10 mg/mL) were added, then mixed thoroughly for 0.5 h. Turbidity was measured before and after mixing, then the mixture was centrifuged to collect the supernatant and determine the absorption spectrum. Different reaction times were also explored for 1 mg/mL curcumin coacervate droplets and 1 mg/mL β-CD. To characterize the morphological changes in curcumin coacervate droplets upon β-CD treatment, we adjusted the pH to 2. After the curcumin coacervate droplets (1 mg/mL) were mixed with β-CD (5 mg/mL), the solution was adjusted to pH 2 and kept for 1 hour. The solution was then adjusted to pH 6, and 50 μL of β-CD (10 mg/mL) was added for an additional 0.5 hours. Next, the above operation was repeated.

### Mechanism of apoptosis induced by curcumin coacervate droplets

For curcumin coacervate droplets-induced toxicity, approximately 1×10⁵ CT26, RAW264.7, and HeLa cells in logarithmic growth phase were inoculated into 96-well plates and cultured overnight in a 5% CO₂ incubator (37 °C). After removing the culture medium, 200 μL of serum-free medium containing different concentrations of curcumin coacervate droplets (0, 0.015, 0.03, 0.06, 0.12, 0.23, 0.45, and 0.9 mg/mL) was added, and the culture was incubated for 4 hours. Six parallel wells were set up in each group. The culture medium was aspirated and washed 3 times with PBS for another 24 h of culture. Then, another 20 μL of CCK-8 solution was added to fresh serum-free medium, incubated for 2 h, and the absorbance was measured at 450 nm using a microplate reader to calculate the cell viability. For β-CD-induced toxicity, only 200 μL of β-CD (2 mg/mL) was added after the PBS wash, and the incubation was continued for an additional 4 h.

CT26 and RAW264.7 cells were cultured as above, and incubated under different conditions (10 and 100 μg/mL of curcumin coacervate droplets for 18 h, 100 μg/mL of curcumin coacervate droplets for 2 and 6 h), then the cells were washed with PBS several times, and observed under a confocal microscope. To assess apoptosis induced by curcumin coacervate droplets, we measured caspase 1 and 4 activity. First, CT26 cells and RAW264.7 cells were incubated with curcumin droplets of different concentrations (0, 10, 20, 30, 50, and 100 μg/mL) for 18 h. After washing the cells with PBS, the protein activity was measured by detecting yellow pNA (p-nitroaniline) produced by caspases 1 and 4. Furthermore, bright-field morphological changes in CT26 cells after incubation with curcumin (100 μg/mL) for 18 h were observed. Finally, after incubating CT26 cells with curcumin coacervate droplets of different concentrations (0, 5, 10, and 15 μg/mL) for 6 h, the cell cycle ratios of CT26 cells were detected using a cell cycle detection kit.

### Release and drug delivery of curcumin coacervates

For the β-CD-induced intracellular release of curcumin coacervate droplets, CT26 cells were incubated with curcumin coacervate droplets (100 μg/mL) for 1 h, washed with PBS several times, and then incubated with β-CD for another 4 h. The cells were observed under a microscope before and after the addition of β-CD. For the delivery of different drugs, different concentrations of cur droplets (0.012, 0.025, 0.05, 0.1, and 0.2 mg/mL), cur/sap (the concentration of sap was 1% of cur), cur/siRNA (the maximum content of siRNA was 0.02 nmol), and cur/DOX (the concentration of DOX was 1% of cur) were first incubated with CT26 cells for 1 hours. The droplets that did not enter the cells were washed away. 2 mg/ml of β-CD was added and incubated for another 4 hours, followed by washing with PBS and incubation for an additional 24 hours. Cell viability was detected by the CCK8 method. Furthermore, the cells' fluorescence was observed under a microscope.

### β-CD-induced antitumor therapy *in vivo*

For the blood compatibility of curcumin coacervate droplets, 1.0 mL of fresh blood from Balb/c mice was collected into EDTA K2 anticoagulation tubes. After removing serum, the precipitates were washed three times with PBS. Cell pelleting was added to 750 μL of PBS or H_2_O containing curcumin droplets (0.05, 0.25, 0.5, and 1 mg/mL) for different incubation times (0.5 and 2 h). Here, PBS and H_2_O were used as negative and positive controls, respectively. After centrifugation (3500 rpm, 10 min), the supernatant was collected for absorbance measurement at 577 nm. Finally, the cell pellet was made into cell smears, stained with Giemsa, and the morphology was observed under the microscope. And the curcumin droplets' hemolysis rate was calculated.

To assess the *in vivo* toxicity of curcumin droplets, 12 five-week-old male BALB/c mice (SPF grade) were randomly divided into 3 groups; 2 groups were killed 1 day after tail vein injection of 200 μL of curcumin droplets (2 mg/mL) or PBS. In contrast, the third groups were killed 15 days after the injection of curcumin droplets. Blood was collected for routine blood and biochemical analyses, and one mouse from each group was used for HE staining.

Approximately 1 × 10^6^ CT26 cells were subcutaneously inoculated into five-week-old male BALB/c mice (SPF grade). 14 days later, 200 μL of the curcumin droplets (2 mg/mL) loaded with Cy5.5 was injected into the tail vein for continuous fluorescence imaging at different times (0.1, 1, 2, 6, 12, 18, and 24 h), and the fluorescence intensity ​​of the localized tumor area was analyzed. At last, the mice were injected with curcumin droplets (2 mg/mL) at different time points (0.1, 1, 2, 6, 12, 18, and 24 h). Blood was collected to measure the fluorescence intensity and analyze the blood half-life of the curcumin droplets.

30 BALB/c mice subcutaneously inoculated with CT26 cells were divided into 6 groups, and treated with (I) PBS, (II) Cur, (III) Cur+β-CD, (IV) Cur/Sap+β-CD, (V) Cur/siRNA+β-CD, (VI) Cur/DOX+β-CD (All droplets were injected through the tail vein, and β-CD was injected with intratumor after 12 h injection of droplets. All the injections were repeated for 3 time every two days). After the corresponding treatment, one mouse was taken from each group. HE, TUNEL, caspase-1, caspase-8 immunofluorescence staining, and autofluorescence were detected with the microscope. For the remaining mice, weight and tumour volume changes were recorded every 2 days using digital vernier calipers and a weight scale. After 14 days, the mice were sacrificed, and the tumours were weighed.

**Statistical analysis.** Experiments were performed with at least three replicates. All values were presented as means ± S.D. Statistical analysis was performed with GraphPad Prism (ver. 8.3.0). Comparison between two groups was performed using a two-tailed Student's t-test. Values with P < 0.05 are considered significant.

## Results and Discussion

### Curcumin forms fluorescent coacervates in aqueous solutions

**Figure 1:**
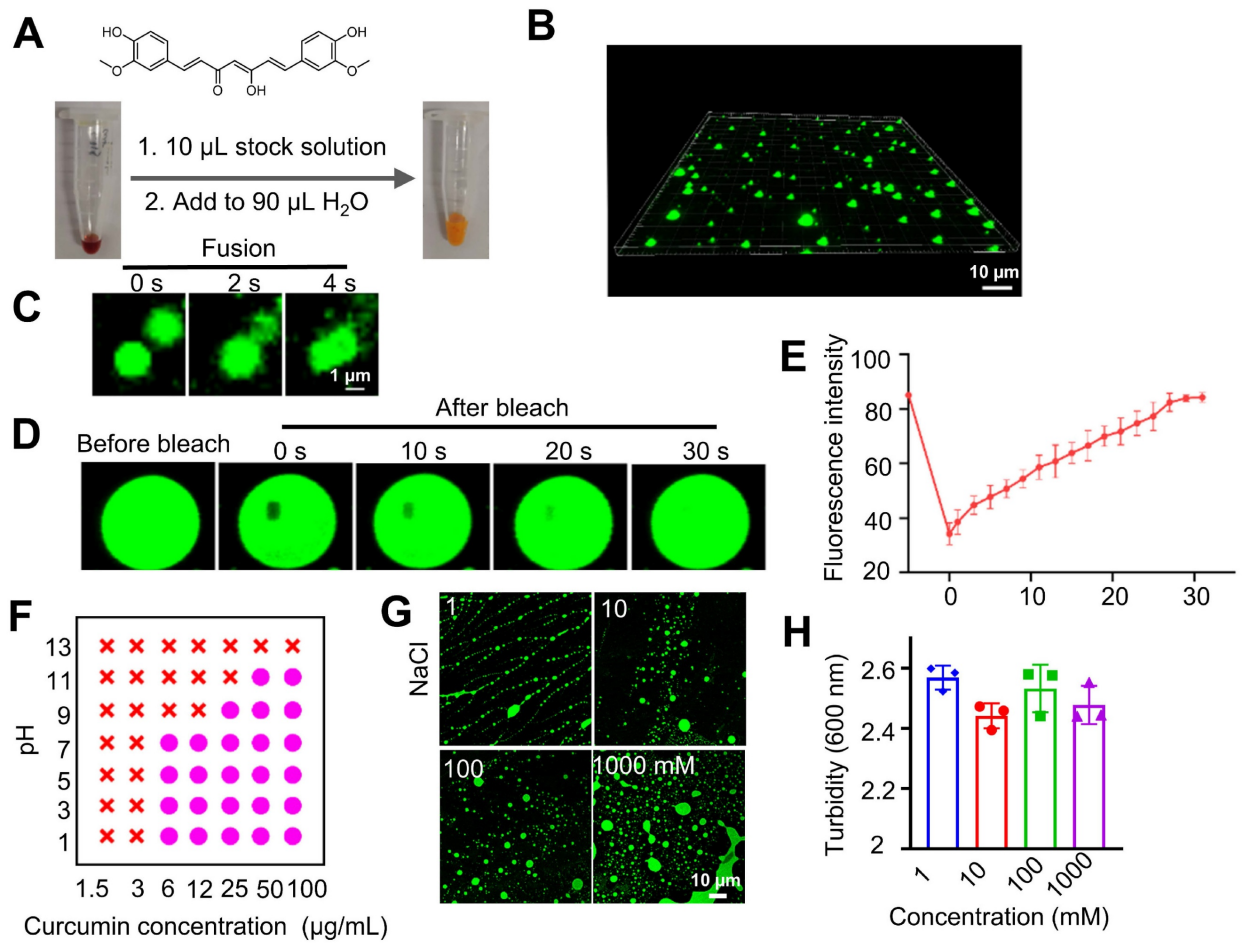
** Curcumin forms fluorescent coacervates in the aqueous solution.** (**A**) Dilution of a curcumin solution in DMSO to water forms fluorescent microdroplets. (**B**) 3D fluorescent microscopic images of the curcumin coacervate droplets. The excitation and emission wavelengths of the fluorescent images were set at 488 nm and 525 nm, respectively. (**C**) Curcumin microdroplets spontaneously fuse with each other. (**D**) The photobleached region of the curcumin droplets spontaneously recovers the fluorescent after 30 seconds. The excitation and emission wavelengths were set at 488 nm and 525 nm, respectively. (**E**) Quantification of the fluorescence recovery after photo-bleaching (FRAP) analysis. (**F**) Phase diagram of curcumin at different pH and concentration conditions. Purple dots indicate conditions of phase separation, and red crosses indicate no coacervate formation. (**G**) Fluorescent microscopic images of curcumin coacervate droplets at different concentrations of NaCl. (**H**) Turbidity of curcumin coacervate droplets at different concentrations of NaCl. Data are presented as mean ± standard deviation (n = 3 independent samples). The excitation and emission wavelengths of the fluorescent images were set at 488 nm and 525 nm, respectively.

Curcumin is a dietary anti-inflammatory and chemopreventive agent consisting of two methoxyphenol rings connected by a conjugated heptadienedione chain. Curcumin's poor water solubility is known to limit its bioavailability and clinical use. Curcumin is well dissolved in DMSO to give a concentrated stock solution. Diluting the stock solution into water incurs the formation of a yellow emulsion in the aqueous solution. Under the confocal microscope, we observed that the emulsion contained fluorescent, globular microdroplets of several microns in diameter (**Figures 1A-B**). Droplet formation correlates with a significant enhancement of fluorescence (**Figure S1**). However, fluorescence decreased at high concentrations of curcumin, likely due to the quenching effect in the concentrated solutions 38. Microdroplets in the aqueous solution can fuse into larger droplets (**Figure 1C**), showing mobility and liquid-like features 39, 40. When a portion of the curcumin droplet was photo-bleached by laser light, the fluorescence gradually recovered to its original strength within 30 seconds (**Figures 1D-E**). All these data show that the droplets are not solid precipitates; instead, they possess the feature of coacervates (also known as condensates) formed due to LLPS. The microdroplets are thereby called curcumin coacervates. Next, we found higher concentrations of curcumin and lower pH promoted LLPS and coacervate formation (**Figures 1F and S2**). High salt concentrations did not diminish coacervate formation (**Figures 1G-H**), suggesting that electrostatic interactions are not the main driving force for coacervation. Curcumin's coacervate-forming property can be explained by its structure, which fits the “stick-and-spacer” mode of phase-separating molecules 13, 14: the two methoxyphenol rings serve as stickers, and the hydrophilic heptadienedione chain serves as the linker. We speculate that a small amount of DMSO may remain in the coacervates, possibly driving the formation of curcumin coacervates in the aqueous phase. However, the exact mechanism of curcumin coacervate formation remains unclear. We also found that when we used ethanol and acetone as solvents for the stock solution, curcumin coacervates could also form in the aqueous solution (**Figures S3-S4**).

### Cyclodextrin induces the dissolution of curcumin coacervates

**Figure 2:**
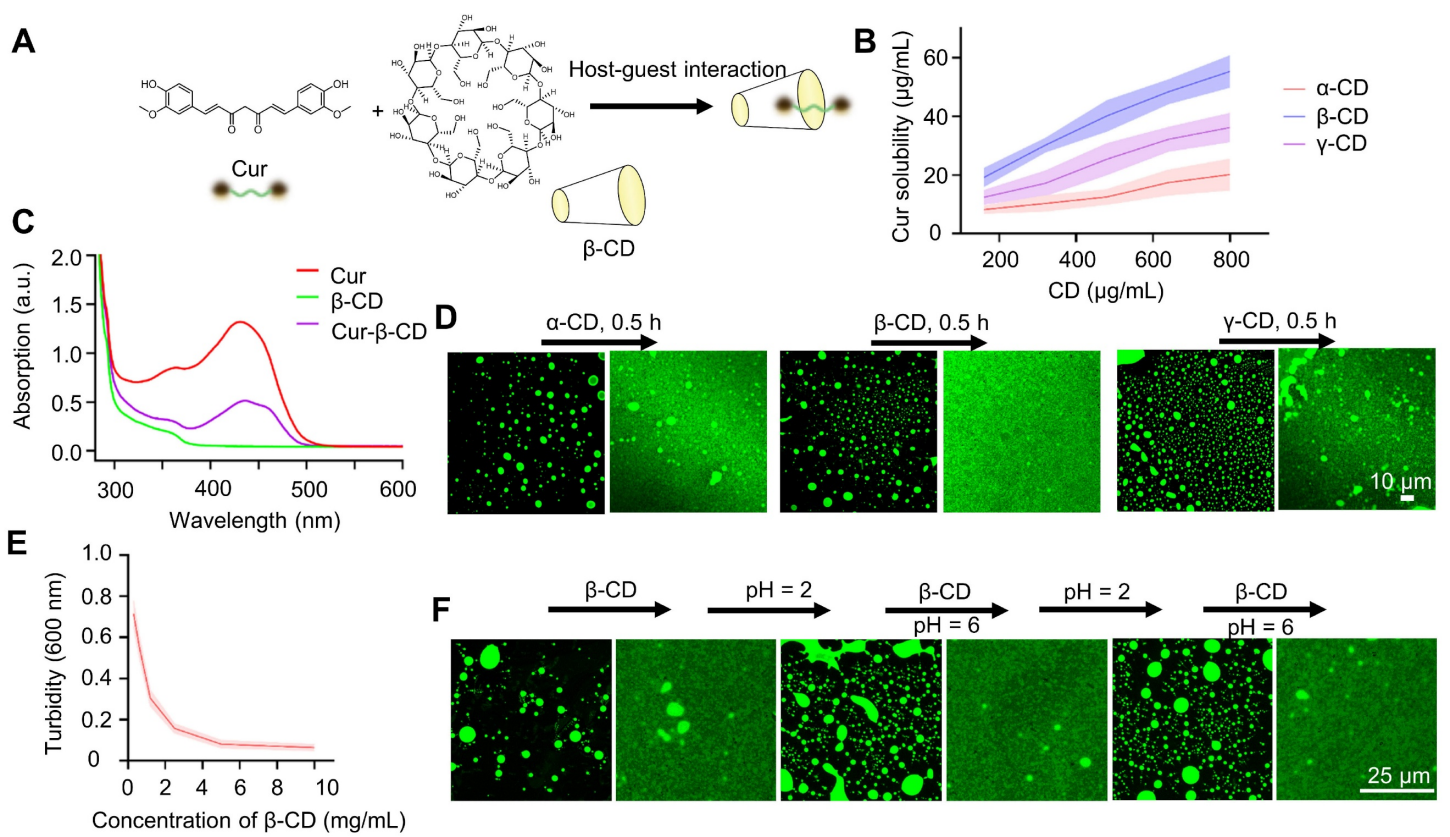
** CD-induced dissolution of curcumin coacervates.** (**A**) Host-guest interaction of curcumin and β-CD significantly changes the hydrophobicity of curcumin. (**B**) Curcumin solubility at different concentrations of cyclodextrins. Briefly, different concentrations of cyclodextrin solution were taken, and 2 mg of curcumin was added to the solution for 0.5 h. Then, after centrifugation, the characteristic absorption value of curcumin at 425 nm was detected to calculate the curcumin concentration according to the standard curve. Standard curve of curcumin UV-vis absorption at 425 nm. Data are presented as mean ± standard deviation (n = 3 independent samples). (**C**) UV-Vis spectra of curcumin-β-CD supramolecular complexes. (**D**) Fluorescent microscopic images of curcumin droplets before and after adding CD. The concentration of curcumin coacervates was 1 mg/mL, and CD was 10 mg/mL. (**E**) Turbidity of curcumin coacervate droplets (1 mg/mL) after adding β-CD for 0.5 h. Data are presented as mean ± standard deviation (n = 3 independent samples). (**F**) Reversible coacervate formation by adding β-CD (5 mg/mL) at pH 6 or adjusting the pH to 2 with 1 M HCl. Data are presented as mean ± standard deviation (n = 3 independent samples). The excitation and emission wavelengths were set at 488 nm and 525 nm, respectively.

The interaction between curcumin and cyclodextrins (CD) is a classical supramolecular interaction 41, 42. We reason that the interaction between curcumin and cyclodextrins will shield the hydrophobicity of the methoxyphenol groups and significantly increase curcumin's water solubility, leading to the dissipation of the coacervates (**Figure 2A**). First, we examined the interactions between α-CD, β-CD, and γ-CD with curcumin based on the solubility of curcumin at different concentrations of CD (**Figure 2B**). At a given concentration, β-CD has a higher capability of solubilizing curcumin, which is consistent with previous reports that β-CD has a stronger interaction with curcumin, whereas all three CDs can interact with curcumin 43. The formation of supramolecular complexes between curcumin and β-CD can also be verified by the generation of new peaks in the UV-Vis and IR spectra (**Figure 2C and S5**). Adding cyclodextrin solutions to the curcumin coacervates caused the dissolution of the microdroplets (**Figure 2D**). Although all three CDs can dissolve the microdroplets, the addition of β-CD gave a complete disappearance of the microdroplets. 5 mg/mL of β-CD significantly decreased the turbidity of 1 mg/mL of curcumin in 30 min (**Figures 2E, S6, and S7**). The supramolecular complex of β-CD and curcumin is also reversible. The acidic condition of pH 2 protonates the β-CD and destroys the supramolecular complexes, leading to the recurrence of the curcumin coacervates. Adding a fresh batch of β-CD at pH 6 again dissolves the coacervates. These cycles can be repeated multiple times (**Figure 2F**). All the evidence above illustrates that β-CD most effectively reverts LLPS of curcumin droplets by interacting with curcumin, causing the dissolution of the coacervates.

### Cargo recruitment in curcumin coacervates

**Figure 3:**
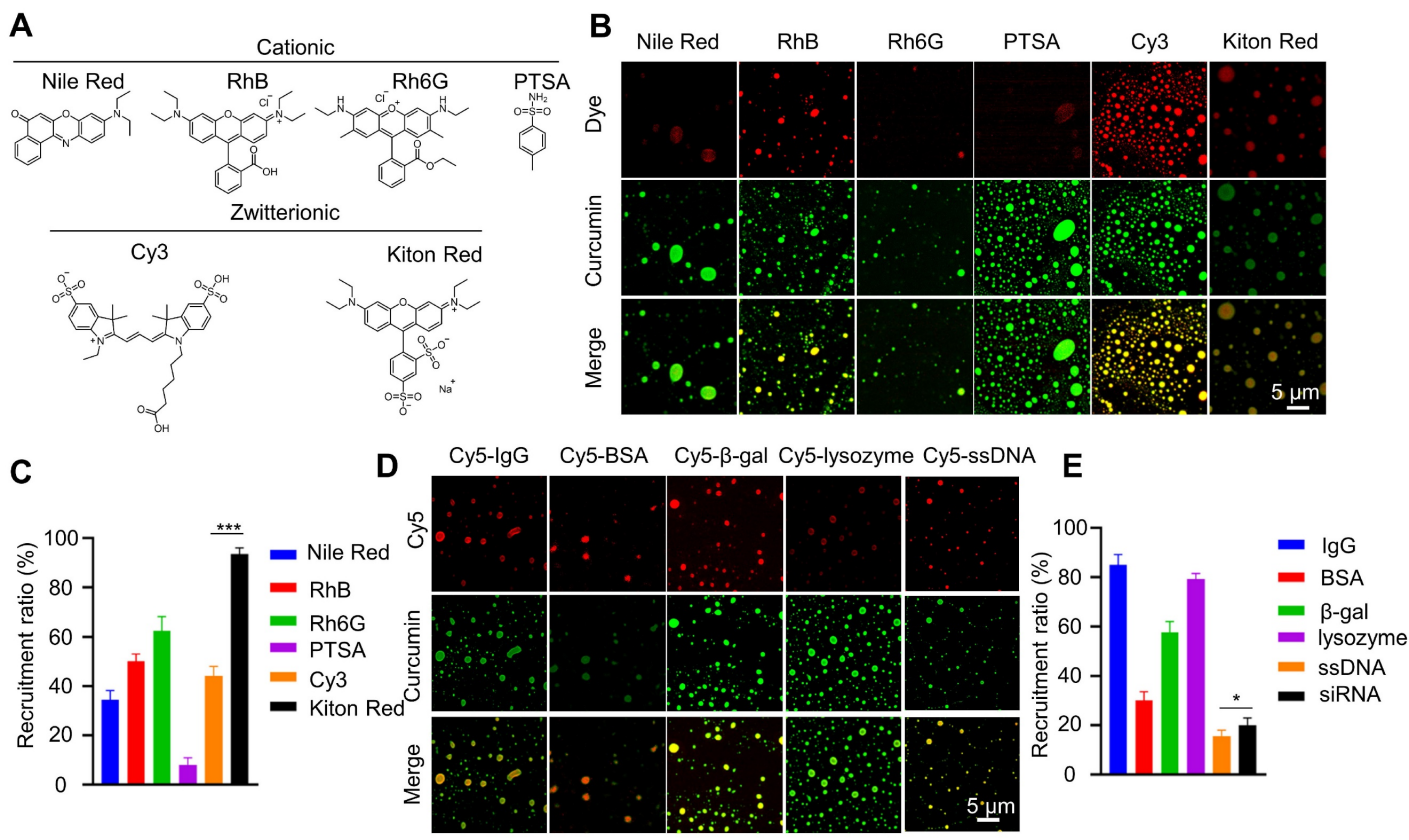
**Recruitment of different molecules in curcumin coacervates**. (**A**) Chemical structures of different fluorescent dyes. (**B**) Fluorescent microscopic images of dye-encapsulated curcumin coacervates. (**C**) Quantification of the recruitment efficiency of fluorescent dyes. The excitation and emission wavelengths were set at 552 nm and 570 nm for the red channel, and 488 nm and 525 nm for the green channel. (**D**) Fluorescent microscopic images of curcumin coacervates with Cy5-labeled antibodies, proteins, enzymes, and nucleic acids. (**E**) Quantification of the recruitment efficiency. Data are presented as mean ± standard deviation (n = 3 independent samples). Curcumin concentration: 10 mg/mL; dye concentration: 0.1 mg/mL. The excitation and emission wavelengths were set at 652 nm and 670 nm for the red channel, and 488 nm and 525 nm for the green channel.

Next, we investigate whether curcumin coacervates can encapsulate molecules, a prerequisite for serving as a vehicle for translocating them into cells. Curcumin coacervates have different recruitment efficiencies for cationic and zwitterionic fluorescent molecules (**Figures 3A-C**). Among zwitterionic molecules, significant differences were found between Kiton red and Cy3, suggesting that molecular structures play an important role. Notably, although the efficiency varies, curcumin coacervates also recruit a range of biomacromolecules such as IgG, proteins, and nucleic acids (**Figures 3D-E**). The recruitment efficiency for ssDNA is lower than that of proteins, possibly because of the negative charges of the nucleic acids. Nevertheless, we observed that in coacervates formed by 1 mg/mL of curcumin, 0.5 nmoL/mL of ssDNA could be encapsulated, which will be sufficient for its bioactivity, and for siRNA, the encapsulation rate of siRNA was around 20%. These data show that curcumin coacervates can serve as vehicles for a range of molecules, including small-molecule dyes, proteins, and nucleic acids.

### Cellular distribution and cellular effects of curcumin coacervates

**Figure 4:**
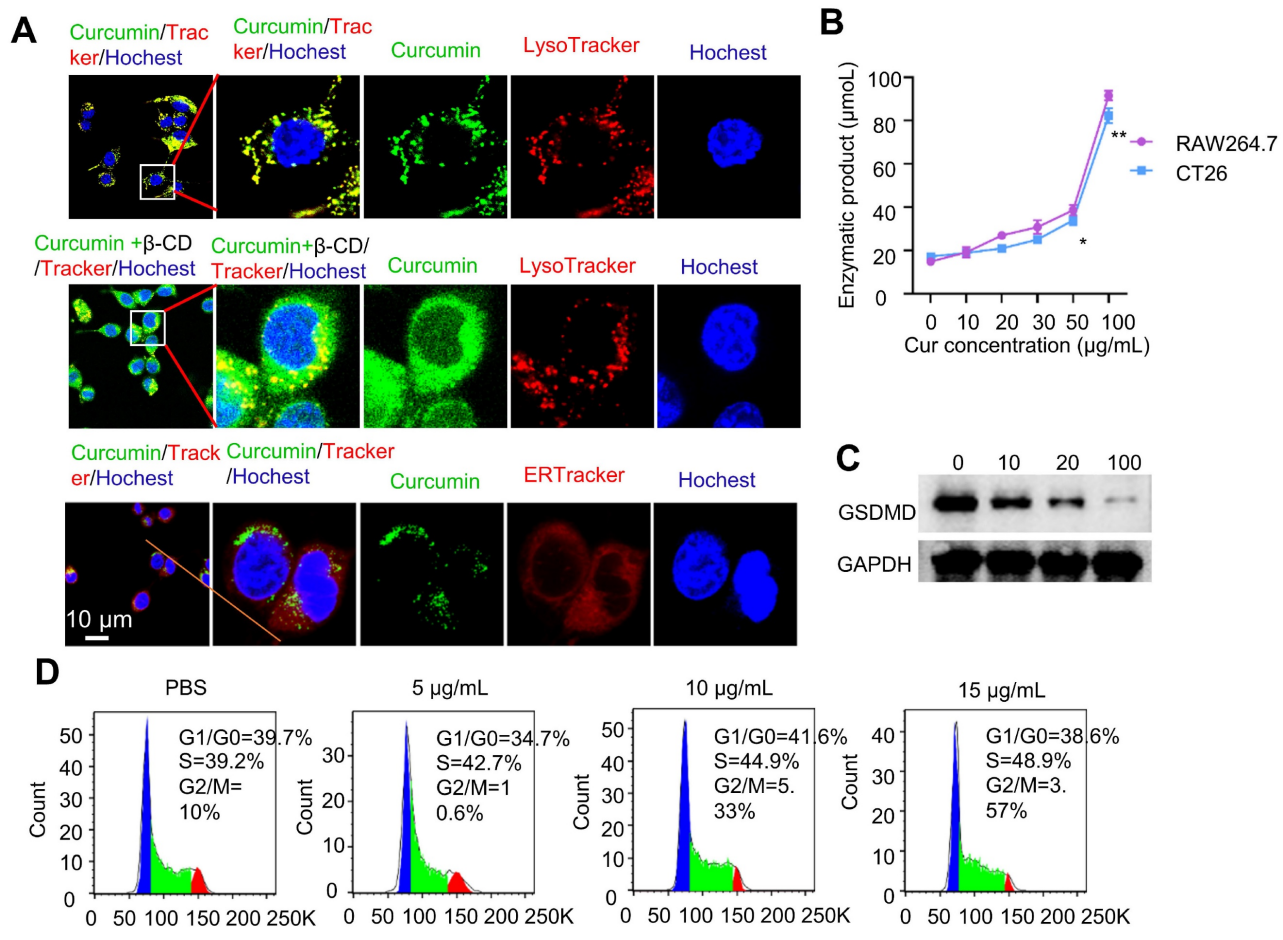
**Curcumin coacervates induce pyroptosis**. (**A**) Fluorescent microscopy images of coacervates with dyes specific for endoplasmic reticulum and lysosomes and after the addition of β-CD. Curcumin coacervates (50 μg/mL) were incubated with CT26 cells together with trackers for 2 h. The excitation and emission wavelengths were set at 652 nm and 670 nm for the red channel, 488 nm and 525 nm for the green channel, and 405 nm and 460 nm for the blue channel, respectively. (**B**) Enzymatic assay showing the increased activity of caspase 1 in cells treated with different concentrations of curcumin coacervates. *: *p* < 0.05; **: *p* < 0.01, compared to the 0 μg/mL of curcumin coacervate group. (**C**) Western blot experiments for GSDMD in CT26 cells 24 hours after treatment with different concentrations of curcumin coacervates. (**D**) Cell cycle changes induced by curcumin coacervates (0, 5, 10, and 15 μg/mL).

Before conducting the intracellular experiments, we first studied the stability of the curcumin coacervate droplets by incubating them with the culture medium containing 10% serum. The results showed that in RPMI 1640 cell culture medium with or without 10% serum, curcumin coacervates exhibit similar droplet morphology (**Figure S8A**). Interestingly, the droplets were significantly smaller in the presence of serum. The concentration-dependent changes in the solution turbidity showed a similar trend with or without serum (**Figure S8B**). Despite the size difference, the droplets remained stable over time in both media (**Figure S8C**). After incubating curcumin coacervates with cells, the droplets were taken up by the cells and shown as puncta inside cells (**Figure 4A**). The fluorescent signal of curcumin coacervates was found to colocalize with that of the LysoTracker but not the ER tracker in cells, suggesting the uptake of curcumin coacervates by the lysosome. Subsequently, after β-CD was added to the cell culture, curcumin coacervates were found to be redistributed into a more expansive space of the cytoplasm, suggesting the release of curcumin coacervates from the lysosome into the cytosol (**Figure 4A**). Further experiments showed Cy5-labeled β-CD alone enters the lysosome (**Figure S9**). These results collectively suggest that β-CD enters the lysosome, disrupts the droplets, and subsequently leads to the leakage of curcumin and the cargo from the lysosome. In addition, β-CD may interact with the lysosomal membrane, altering its fluidity or structural integrity and thereby permitting the release of entrapped contents. However, the exact molecular mechanism of this process is currently elusive.

We also observed that curcumin coacervates showed modest cytotoxicity following 1-h incubation at 1 mg/mL of curcumin. Based on the CCK8 assay, elongating the incubation time to 4 h, at the curcumin concentration of 0.45 mg/mL, the survival rates of CT26, RAW264.7, and HeLa cells were all close to 70% (**Figure S10**). After corresponding concentrations of β-CD were added 30 min after the incubation of curcumin (a total incubation time of 4 h), the cell survival rate for CT26 cells was significantly reduced (p < 0.05), possibly because the dissolution of curcumin coacervates releases free curcumin (**Figure S11**). After further elongating the incubation time to 6 h, CT26 cells started to show vacuolation in the cytoplasm. After 18 h, a significant number of vacuoles were observed in the center of the cells (**Figure S12**). These observations are consistent with the characteristics of cell pyroptosis. Next, we used a caspase-1 activity kit to quantify the activity of caspase 1 in cells. Higher concentrations of curcumin coacervates generated higher levels of caspase 1 activity in both CT26 and RAW264.7 cells (**Figure 4B**), but the activities of caspase 4 did not significantly increase with the increase of the curcumin concentration (**Figure S13**). Detecting the cleavage of the gasdermin D protein (GSDMD) provides further evidence of pyroptosis. We examined GSDMD expression in CT26 cells following various treatments. The western blot results showed that after 24 hours of treatment, 100 µg/mL curcumin significantly reduced GSDMD expression in CT26 cells (**Figure 4C**). The amount of LDH released into the extracellular space by CT26 cells treated with increasing concentrations of curcumin coacervates gradually increased (**Figure S14A**). Similarly, in response to the increasing curcumin coacervates, CT26 cells released increasing concentrations of IL-1β (**Figure S14B**). Taken together, these results suggest that curcumin coacervates could induce pyroptosis in CT26 cells. Considering the phagocytosis of curcumin by macrophages, we next performed flow cytometry analysis of CD86 and CD206, and ELISA analysis of IL-1β, TNF-α, and IL-6. It was found that curcumin did not induce macrophage polarization, and no significant polarization occurred with changes in concentration (**Figure S15**). For cytokine secretion, IL-1β changed significantly; this may be because the GSDMD pores formed during pyroptosis promote the release of IL-1β. In contrast, changes in IL-6 and TNF-α were negligible (**Figure S16**), suggesting that curcumin droplets might not elicit severe immunogenic reactions. Under the microscope, we also observed vesicle formation, swelling, and rupture of the CT26 cells (**Figure S17**), consistent with the typical pyroptosis. Cell cycle analysis based on the flow cytometry data showed that higher concentrations of curcumin coacervates correspond to a higher S phase, showing an inhibition of cell proliferation (**Figure 4D**). All these data show that curcumin coacervates did not elicit acute cytotoxicity during a short incubation (1-2 h), but did induce pronounced pyroptosis during a long incubation in cancer cells (18 h or longer).

### Coacervate-mediated delivery of cytotoxic compounds

**Figure 5:**
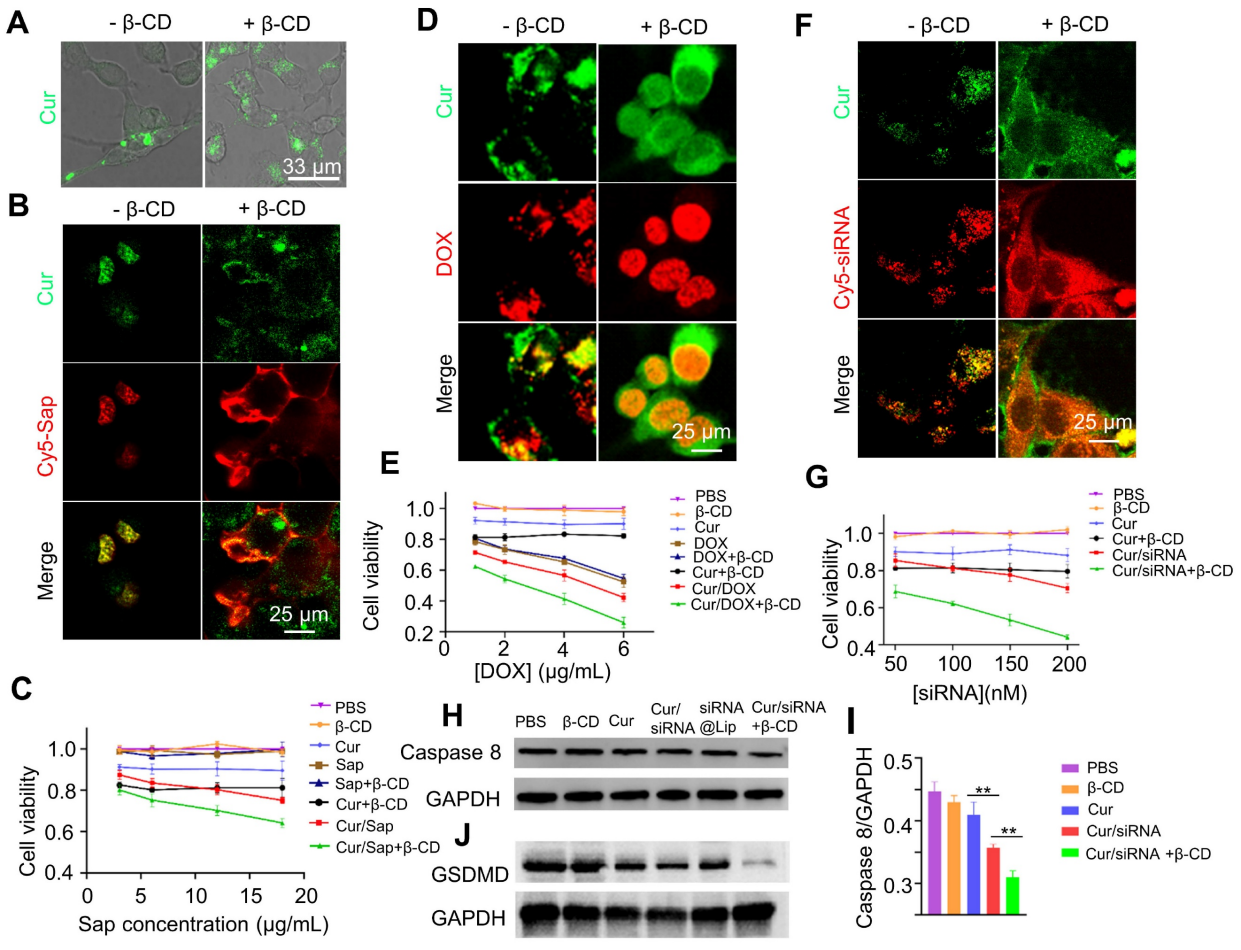
** Delivery and intracellular release of cytotoxic molecules by curcumin coacervates**. (**A**) Fluorescence imaging of CT26 cells incubated with curcumin coacervate droplets (100 μg/mL, 1 h) before and after adding β-CD (1 mg/mL). Briefly, the excitation and emission wavelengths of the fluorescent images were set at 488 nm and 525 nm. (**B, D, and F**)**.** Release of Sap, DOX, and siRNA after adding β-CD (1 mg/mL) to CT26 cells incubated with drug-loaded curcumin coacervates (100 μg/mL). The excitation and emission wavelengths of the fluorescent images were set at 652 nm and 670 nm for the red channel and 488 nm and 525 nm for the green channel. (**C, E, and G**) CCK-8 cytotoxicity of Sap, DOX, and siRNA-treated cells. Curcumin droplets: 0.1 mg/mL; β-CD: 1 mg/mL. (**H**) Western blot data of caspase 8 down-regulation by siRNA 48 hours after cells were treated with different formulations at 37 °C. (**I**) Quantitative analysis of data in (**H**). (**J**) Western blot data of GSDMD 24 hours after cells were treated with different formulations at 37 °C.

We next explored whether curcumin coacervates can deliver bioactive pharmaceuticals into cells. After curcumin coacervates (0.1 mg/mL) were incubated with CT26 cells for 1 h, fluorescent puncta were found to distribute in the cytoplasm (**Figure 5A**). The addition of β-CD (1 mg/mL) to the cell culture led to the re-distribution of the fluorescent signals to a large space of the cytoplasm, suggesting that β-CD dissolves the coacervates and may result in the cytosolic distribution of the cargo.

Next, we investigated whether cytotoxic agents delivered by curcumin coacervates can kill cancer cells. We first delivered saporin (Sap), a plant-derived, non-cell-permeable small protein toxin 44-46. Killing cells by blocking protein synthesis, Sap needs to be located in the cytoplasm to take effect. Curcumin coacervates enriched Cy5-labeled Sap and delivered it into cells. The addition of β-CD to the cell culture dispersed the Cy5-Sap signal to a wider region of the cytoplasm (**Figure 5B**), suggesting a cytosolic release from the coacervates. Cell viability tests revealed the highest cytotoxicity of Sap in the presence of curcumin coacervates and β-CD (**Figure 5C**). Notably, curcumin itself also causes a certain degree of cytotoxicity.

Next, we examined whether curcumin coacervate-mediated delivery can increase the cytotoxicity of the small-molecule anticancer drug doxorubicin (DOX) by enhancing its intracellular uptake. DOX is a widely used anti-tumor drug extracted from *Streptomyces peucetius* 47, 48. Although DOX can spontaneously enter cells and be enriched in the nuclei, encapsulation in curcumin coacervates significantly enhanced the cellular uptake of DOX. Also, we observed a more homogenous distribution of DOX in the cell nuclei in the presence of β-CD (**Figure 5D**). The highest cytotoxicity was also observed in the combination of DOX with curcumin coacervates, which was enhanced by β-CD treatment (**Figure 5E**).

The third cytotoxic drug we chose was a Caspase 8 small interfering RNA (siRNA). siRNA molecules are short double-stranded RNA molecules, usually 20-25 nucleotides in length. siRNA binds to specific mRNA and triggers RNA interference (RNAi), thereby specifically degrading the target mRNA and inhibiting the expression of the targeted genes 49, 50. Although conceived as promising therapeutics, siRNA requires a vehicle for intracellular delivery. Currently, research on siRNA delivery primarily focuses on nanocarriers 51-53. On another note, caspase 8, a member of the cysteine-aspartic acid protease family, interacts with pro-caspase 1 and affects its activation process; when Caspase 8 is inhibited, cell death *via* the Caspase 1-mediated pyroptosis pathway will increase (**Scheme S1**). We examined the serum stability of siRNA encapsulated in curcumin coacervates. We also show that siRNA loaded in curcumin coacervates remained intact for at least 24 hours in cell culture medium (**Figure S18**). Subsequently, we observed that fluorescently labeled Caspase 8 siRNA (Cy5-siRNA) can be delivered by curcumin into cells, and the addition of β-CD significantly improved its intracellular distribution (**Figure 5F**). From the cytotoxicity data, we also found that the addition of β-CD significantly unleashed the cytotoxicity of the Caspase 8 siRNA (**Figure 5G**). For siRNA release, β-CD was added, and after centrifugation at different times, the supernatant was collected to measure siRNA release. The results showed that upon the addition of β-CD, siRNA was gradually released from curcumin coacervates (**Figures S19**). Western blotting analysis also showed a marked knockdown of Caspase 8 (**Figures 5H-I**). Compared with the curcumin group, the cur/siRNA treatment resulted in decreased GSDMD protein expression; this decrease was further reduced upon addition of β-CD. These results demonstrate the combined induction of pyroptosis. The Cur/siRNA+β-CD group showed a more significant suppression of GSDMD than the siRNA@Lip group (**Figure 5H**). In addition, we performed quantitative PCR to measure mRNA levels. The results showed that the Cur/siRNA+β-CD treatment group showed a drastic decrease of around 70% (**Figures S20**). Taken together, we manifested the intracellular delivery of three pharmaceuticals by curcumin coacervates. We demonstrated that β-CD mediated the intracellular re-distribution of the cargo from the coacervates to the cytosol and enhanced their cytotoxicity to cancer cells.

### Curcumin coacervates enrich in the tumor site through tail vein injection

**Figure 6:**
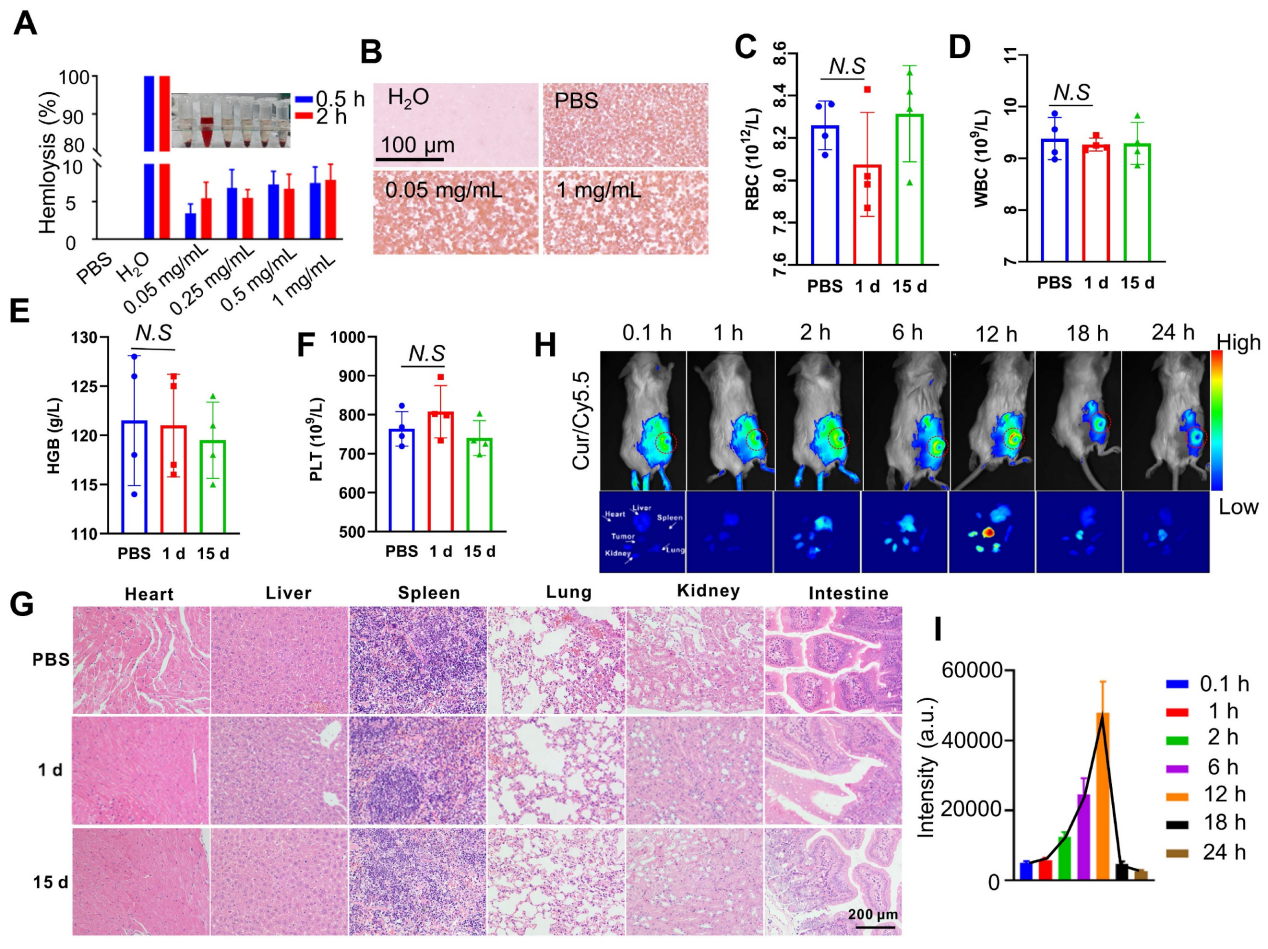
** Blood compatibility and tumor-specific localization of curcumin coacervates via intravenous administration**. (**A**) Hemolytic activity of curcumin coacervates at different concentrations. Briefly, after coacervates were incubated with RBCs, the UV-Vis absorption at 577 nm of the supernatants was measured. The hemolysis rate was measured according to the following: (OD_experiment group_-OD_PBS_)/(OD_water_-OD_PBS_). (**B**) Giemsa staining images of RBCs. (**C-F**) Blood analysis of normal mice after curcumin coacervate droplets injection: RBC, WBC, PLT, and HGB. (**G**) The HE staining results of the corresponding organs after 15 d of PBS injection and 15 d of curcumin coacervate droplets. (**H**) Fluorescent images of subcutaneous CT26 tumor-bearing BALB/c mice and major organs at different time points after tail-vein injection of curcumin coacervates. (**I**) Quantification of the fluorescence intensity at tumor sites according to the images. Briefly, 200 μL of the curcumin droplets (2 mg/mL) loaded with Cy5.5 (0.2 mg/mL) was injected into the tail vein, and the fluorescence intensity (670 nm/700 nm) ​​of the localized tumor area was analyzed with ImageJ.

Next, we investigate the administration route of curcumin coacervates and their distribution *in vivo* in tumor-bearing BALB/c mice. First, we examined the blood compatibility of curcumin coacervates in red blood cell (RBC) hemolysis experiments. Ultrapure water causes complete rupture of RBCs due to the osmotic effect, and phosphate buffer saline (PBS) keeps RBC morphology. Therefore, these two conditions were set as the positive and negative controls. Curcumin coacervates up to 1 mg/mL with an incubation time of 2 h did not cause a significant level of hemolysis (**Figure 6A**). Giemsa staining experiments show a regular morphology and color of the RBCs (**Figure 6B**), suggesting the coacervates are safe for intravenous (i.v.) injections. We then injected 200 μL of curcumin coacervates at 2 mg/mL through i.v. route to healthy BALB/c mice and harvested the blood after 15 days. Blood biochemical analysis showed no significant difference in white blood cells (WBC), RBC, hemoglobin (HGB), and platelets (PLT) (**Figures 6C-F**). Alanine aminotransferase (ALT) and aspartate aminotransferase (AST) levels were also within the normal range (**Figures S21**), indicating that curcumin coacervates had no significant adverse effect on the liver function of mice. Hematoxylin and eosin (HE) staining of the major organs also did not show noticeable changes (**Figure 6G**). These data show that although curcumin coacervates manifested mild cytotoxicity at the cell level, they were well tolerated via i.v. injection: no pathological lesions in organs and tissues were observed.

Next, we injected curcumin coacervates through tail vein injection into mice bearing subcutaneous CT26 tumors. Curcumin coacervates were labeled with a Cy5.5 dye, allowing us to track the distribution of the microdroplets *in vivo*. Whole mouse fluorescent imaging revealed a gradual increase in the fluorescent signal at the tumor site 2 hours after curcumin coacervate injection, peaking at 12 h before attenuation, a finding also confirmed by organ-specific fluorescent imaging. (**Figure 6H**). Quantification of the fluorescence intensity also verified this trend (**Figure 6I**). Enrichment of coacervates at the tumor site through i.v. injection has been previously reported 14 and is likely due to the enhanced permeability and retention effect 54. By analyzing the amount of Cy5.5 fluorescence in the blood, we found curcumin coacervates had a half-life of 3 hours in the mouse circulation system (**Figure S22**), significantly longer than the reported half-life of the curcumin molecule *in vivo* (1-2 hours) 55. These data support the delivery of anticancer therapeutics by curcumin coacervates *via* i.v. injection.

### Curcumin coacervates deliver siRNA for enhanced pyroptosis *in vivo*

**Figure 7:**
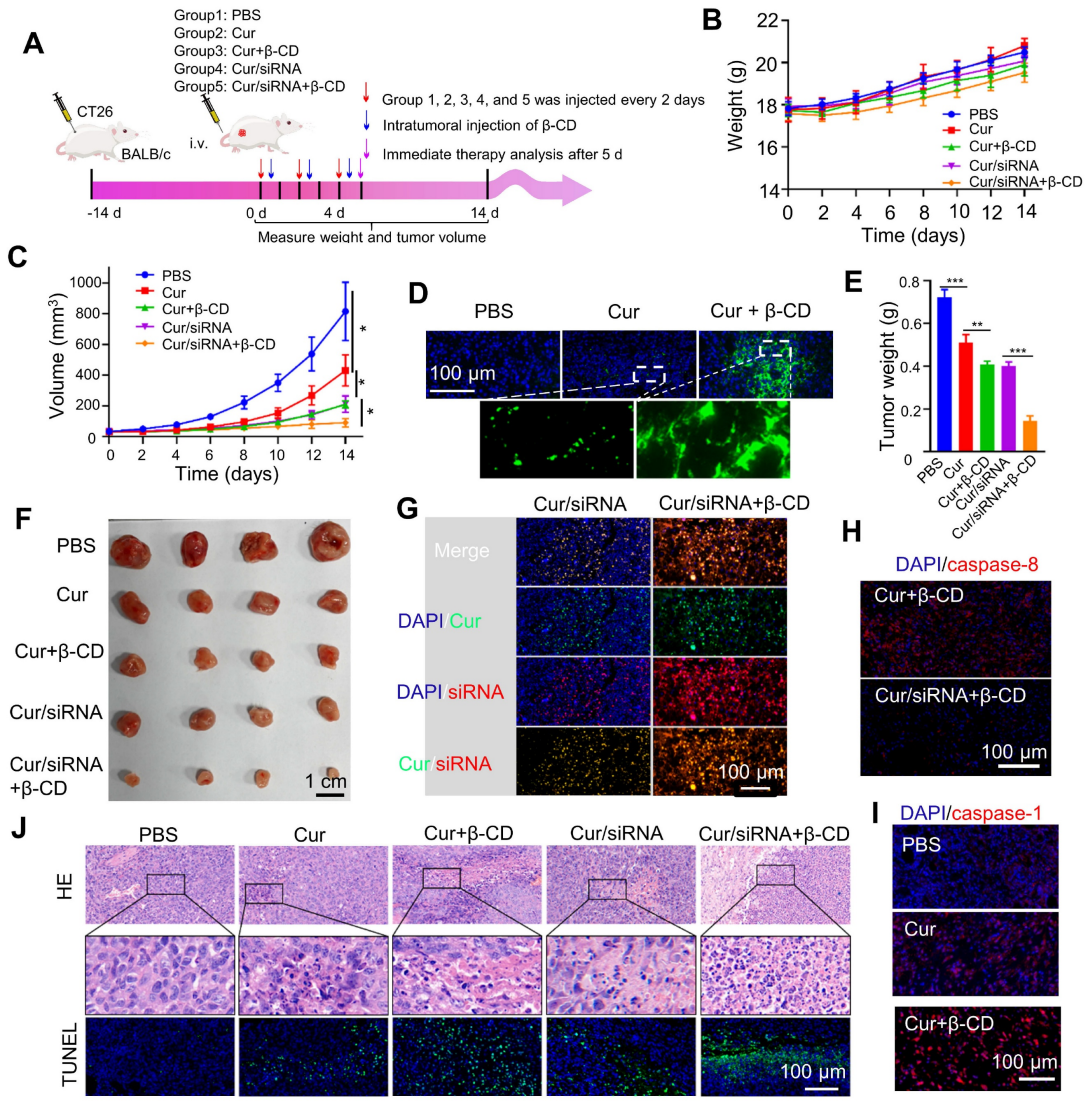
** siRNA-loaded curcumin coacervates for enhanced pyroptosis *in vivo*.** (**A**) Schematic illustration showing the timeline of the animal experiment. Briefly, 1×10^6^ CT26 cells were subcutaneously inoculated onto the back of BALB/c mice. When tumors grew to about 40 mm^3^, 200 μL of the coacervate solutions (curcumin: 2 mg/mL, siRNA: 1nmol/mL) were injected through the tail vein. After 12 hours, 50 μL β-CD (10 mg/mL) was injected into the tumor in the selected groups. The above operation was performed every two days and repeated three times. The sizes of the tumor were measured by vernier calipers. (**B**) Comparison of the body weights of animals during the treatment period. (**C**) Comparison of tumor volumes monitored during the treatment period. (**D**) Fluorescent images of tumor slices showing the enrichment of curcumin coacervates in the tumor tissue and β-CD-induced enhancement of the curcumin fluorescence. (**E**) Quantification of dissected tumor weights after 14 days. (**F**) Images of tumor tissues dissected at day 14 in each group (four animals in each group). (**G**) Fluorescent images of tumor slices showing the presence of curcumin and fluorescently labeled siRNA in the tumor tissues, and that β-CD enhanced the enrichment of curcumin and siRNA. (**H**) Immunofluorescent staining of caspase 8 showing siRNA-loaded curcumin coacervates significantly decreased caspase 8 level in the tumor slices. (**I**) Immunofluorescent staining of the slices showing an increase of caspase 1 in the treatment groups that received siRNA-loaded curcumin coacervates and β-CD treatment. (**J**) HE and TUNEL-stained images of tumors in different treatment groups showing signs of cell death. Briefly, 5 d after the treatment, the mouse tumors were surgically removed, fixed with paraformaldehyde, embedded in paraffin, stained and photographed. Data are presented as mean ± standard deviation (n = 4 independent samples). Two-sided student's t-test, ** *P* < 0.01, *** *P* < 0.001.

Lastly, we evaluated the delivery of anticancer siRNA by curcumin coacervates *in vivo* and the anticancer treatment to tumor-bearing mice. CT26 tumor-bearing BALB/c mice were injected three times with curcumin coacervates in the absence or presence of caspase 8 siRNA by tail vein injections, and 12 hours after every coacervate injection, β-CD was introduced to the tumor cells *via* intratumoral injections (**Figure 7A**). We evaluated the therapeutic efficacy after 14 days. Mice in all the treatment groups showed similar trends of body weight changes (**Figure 7B**), showing that curcumin coacervates plus β-CD injection did not cause significant toxicity to mice. By monitoring the changes in tumor volume, we found that the tumors in the PBS group grew the fastest, while curcumin coacervate treatment slightly suppressed tumor growth (*p* < 0.05) (**Figure 7C**), showing that curcumin coacervates enriched in the tumor sites produced local toxicity to CT26 cells. Furthermore, adding the intratumoral injection of β-CD further suppressed tumor growth, possibly because β-CD-induced destruction of curcumin coacervates released free curcumin into the cytosol and caused cell death in the tumor tissue. Tissue slices showed that curcumin coacervates fluorescently labeled tumor tissues, shown as granular puncta, whereas the intratumoral injection of β-CD diffused the fluorescent signal (**Figure 7D**), consistent with β-CD-induced dispersion of curcumin coacervates *in vivo*. Mice that received i.v. injection of siRNA-loaded coacervates, followed by intratumoral injection of β-CD, exhibited the most pronounced tumor shrinkage (**Figures 7C**,** 7E, and 7F**). β-CD treatment significantly increased the distribution of siRNA in tumor tissues (**Figure 7G**), presumably by releasing siRNA from coacervates in cancer cells. Subsequently, we demonstrated evidence of the decreased expression of caspase 8, the concomitant increase of active caspase 1, and the cleavage of GSDMD protein (**Figures 7H and 7I, Figure S23**). HE and TUNEL staining results showed evidence of death (**Figure 7J**), which we believe is the direct reason for tumor shrinkage. Taken together, here we show *in vivo* evidence that curcumin coacervates, combined with β-CD-assisted intracellular release, serve as an effective strategy for siRNA delivery. This coacervate/siRNA/β-CD formulation may become a new anticancer therapy (**Scheme 1**).

## Conclusion

Curcumin, derived from the rhizome of Curcuma longa, is one of the primary ingredients in turmeric and curry powders that are used as spices in Middle Eastern and Asian countries, especially on the Indian subcontinent. Besides being used as a dietary supplement, curcumin is also a Chinese medicine ingredient that exhibits a wide range of activity for human pathological conditions. In particular, curcumin is known for its anti-inflammation and antioxidation efficacy, as well as antitumor and immune regulation activities 56, 57. Yet, why curcumin exerts such a wide range of biological activities remains elusive. Curcumin was reported to inhibit liquid-liquid phase separation of disease-associated proteins such as fused in sarcoma (FUS) and α-Synuclein condensates 58, 59. Due to the extremely low water solubility of free curcumin molecules, researchers often use carriers to facilitate its delivery for *in vivo* applications. For example, liposomes, niosomes, polymeric nanoparticles, dendrimers, gold NPs, carbon nanotubes, and other NP formulations were used to facilitate the pharmaceutical use of curcumin 57. Notwithstanding, it has not been conceived that curcumin may form phase-separated coacervates, which can be used as siRNA carriers. Here, we report a previously unnoticed property of curcumin. Under certain conditions, curcumin solutions may form phase-separated coacervates in aqueous solution, which can encapsulate biological molecules such as siRNA into cells. Cyclodextrins, especially β-CD, can disintegrate curcumin coacervates and release the cargo into the cytoplasm. We have shown that curcumin coacervates did not elicit acute cytotoxicity during a short incubation (1-2 h), but did induce pronounced pyroptosis during a prolonged incubation (18 h or longer). Furthermore, we demonstrate that curcumin coacervates can be used as drug carriers to deliver cytotoxic agents into cancer cells, and β-CD-mediated re-distribution of the cytotoxic agents into the cytoplasm increases the enhanced pyroptosis. We showcase the anticancer potential of the curcumin/siRNA formulation using an siRNA that binds to the mRNA of caspase 8 and knocks down caspase 8 (**Scheme S1**). siRNA-loaded curcumin coacervates exhibited marked anticancer activity both *in vitro* and in tumor-bearing BALB/c mice, and β-CD injection further enhances the tumor-killing effect. Impressively, intravenous injection of curcumin coacervates into the blood circulation of tumor-bearing BALB/c mice resulted in selective enrichment of the coacervates in tumor tissues, enabling tumor-targeted delivery via systemic administration. In this study, we employed intratumoral injection of β-CD following administration of curcumin coacervate/siRNA mixture. This procedure is designed to achieve a high concentration of β-CD at the tumor site, leading to the disintegration of curcumin coacervates within cancer cells and the release of siRNA into the cytoplasm, where it can exert its action. In the future, intravenous injection of β-CD post administration of the coacervates may be explored.

Canonical carriers of nucleic acid drugs, such as nanoparticles or liposomes, have a clearly defined boundary that separates the interior of the particle from the outside. In contrast, curcumin coacervates are highly dynamic. The dynamicity of coacervates is reflected at least in three aspects: (1) molecules inside coacervates constantly exchange with molecules in the bulk solution, yet maintaining a stable droplet entity, (2) the coacervates have a fluid-like feature, and (3) coacervates may fuse into larger droplet, or reform into smaller droplets in particular biological environment (for example, in the presence of serum protein, curcumin droplets became smaller). All these features show that although small molecule coacervates resemble nanoparticles in many aspects, they might adopt a different mechanism of drug delivery. At present, the mechanisms underlying curcumin coacervate formation, their endocytosis pathway, and their tumor-specific targeting remain elusive. Research aimed at answering these questions is currently underway.

Taken together, we report that the TCM compound curcumin possesses a unique property to form coacervates, which can serve as a carrier for biologically active compounds through LLPS and coacervate formation. Utilizing this property, we demonstrate tumor-targeted delivery of caspase 8 siRNA for cancer treatment in animal models, showing the potential of the curcumin/siRNA formulation as a new strategy for cancer treatment and the importance of using the β-CD-curcumin supramolecular interaction to induce enhanced pyroptosis.

## Supplementary Material

Supplementary figures.
