# Supplementary material for: Curcumin coacervates for supramolecular-interaction-responsive cytosolic siRNA delivery to enhance pyroptosis
Source: Theranostics. 2026 Jan 1;16(6):2705–20. doi: 10.7150/thno.121865 (PMC12775651; doi:10.7150/thno.121865)
Supplement: Supplementary file 1 — Supplementary figures. [file thnov16p2705s1.pdf]

Supporting Information

Curcumin Coacervates for Supramolecular-Interaction-Responsive Cytosolic siRNA Delivery to Enhance Pyroptosis

Kai Cheng,[a,†] Fang Zhang,[b,†] Yishu Bao,[a] Zhiyi Xu,[a] Hao Kong,[a] Dingdong Yuan,[a] Zhong Zheng,[a] Yuan-Di Zhao,*,[b] Jiang Xia*,[a]

[a] Department of Chemistry, The Chinese University of Hong Kong, Shatin, Hong Kong SAR, China.

[b] Britton Chance Center for Biomedical Photonics at Wuhan National Laboratory for Optoelectronics-Hubei Bioinformatics & Molecular Imaging Key Laboratory, Department of Biomedical Engineering, College of Life Science and Technology, Huazhong University of Science and Technology, Wuhan 430074, Hubei, P. R. China

*Address correspondence to zydi@mail.hust.edu.cn (Y.D. Z.); jiangxia@cuhk.edu.hk (J. X.) ORCID 0000-0001-8112-7625

Phone: (852) 3943 6165

Fax: (852) 2603 5057

† K. C. and F. Z. contributed equally.

Contents

Item	Page No.
**Figure S1.** Fluorescence spectra of curcumin dissolved in DMSO and DMSO/H2O at different concentrations.	**S4**
**Figure S2.** Characterization of curcumin coacervates.	**S5**
**Figure S3.** Curcumin coacervates derived from a different organic solvent, ethanol.	**S6**
**Figure S4.** Curcumin coacervates derived from a different organic solvent, acetone.	**S7**
**Figure S5.** UV-Vis and IR spectra of curcumin-β-CD supramolecular complexes.	**S8**
**Figure S6.** Turbidity of curcumin coacervates after the addition of β-CD.	**S9**
**Figure S7.** UV-Vis spectra of curcumin-β-CD supramolecular complexes at different concentrations of β-CD.	**S10**
**Figure S8.** Curcumin coacervates in cell culture medium.	**S11**
**Figure S9.** Fluorescent microscopy images of Cy5-β-CD co-stained with lysosomes.	**S12**
**Figure S10.** Cytotoxicity of curcumin coacervates in CT26, RAW264.7, and HeLa cells measured based on the CCK-8 assay.	**S13**
**Figure S11.** Cytotoxicity of curcumin coacervates in CT26, RAW264.7, and HeLa cells measured based on the CCK-8 assay before and after the addition of β-CD.	**S14**
**Figure S12.** Fluorescent microscopy images of CT26 cells after incubation with the curcumin coacervates.	**S15**
**Figure S13.** Caspase 4 activity measurement based on the colorogenic reaction of a caspase 4 substrate of curcumin-coacervate-treated cells.	**S16**
**Figure S14.** Measurement of LDH and IL-1b from curcumin-coacervate-treated cells.	**S17**
**Figure S15.** Flow cytometry data of curcumin-coacervate-treated RAW264.7 macrophages.	**S18**
**Figure S16**. ELISA-based measurement of the secretion of IL-1β, IL-6, and TNF-α from curcumin-coacervate-treated RAW264.7 macrophages.	**S19**
**Figure S17**. Morphology changes of curcumin-coacervate-treated CT26 cells.	**S20**

**Figure S18.** Stability of double-stranded siRNA with or without coacervates in cell culture medium.	**S21**
**Figure S19.** β-CD promotes the release of siRNA from curcumin coacervates.	**S22**
**Figure S20**. mRNA level of Caspase 8 in CT26 cells after 24-hour treatment with different formulations.	**S23**
**Figure S21.** Quantification of the liver function indicators ALT and AST.	**S24**
**Figure S22.** Measurement of the circulating curcumin coacervates in the blood.	**S25**
**Figure S23**. Tumor tissue slides stained for GSDMD showing signs of pyroptosis.	**S26**
**Scheme S1.** Schematic illustration showing the mechanism of action of caspase 8 siRNA for cancer treatment.	**S27**
**The uncropped western blot images**.	**S28**

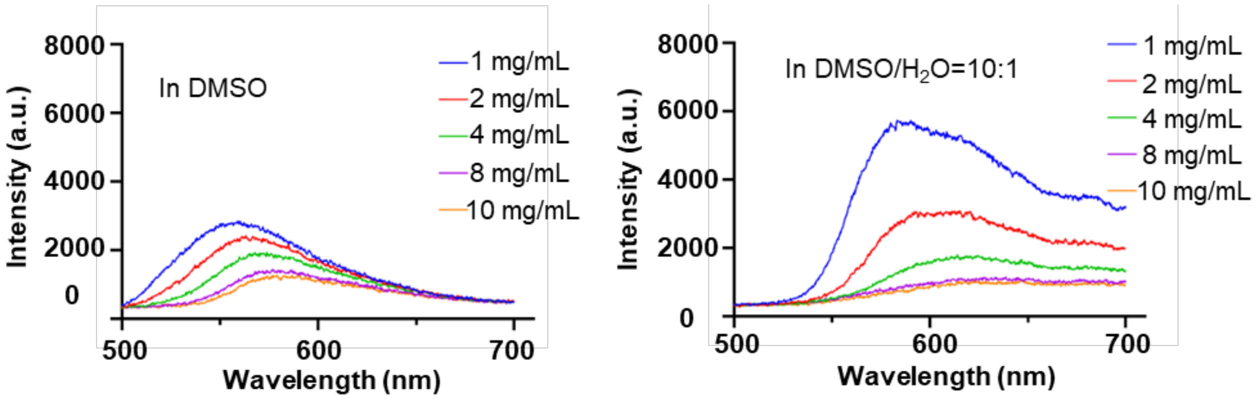

Figure S1. Fluorescence spectra of curcumin dissolved in DMSO and DMSO/H_2O at different concentrations. Briefly, the excitation wavelength for the fluorescent spectra was set to 425 nm.

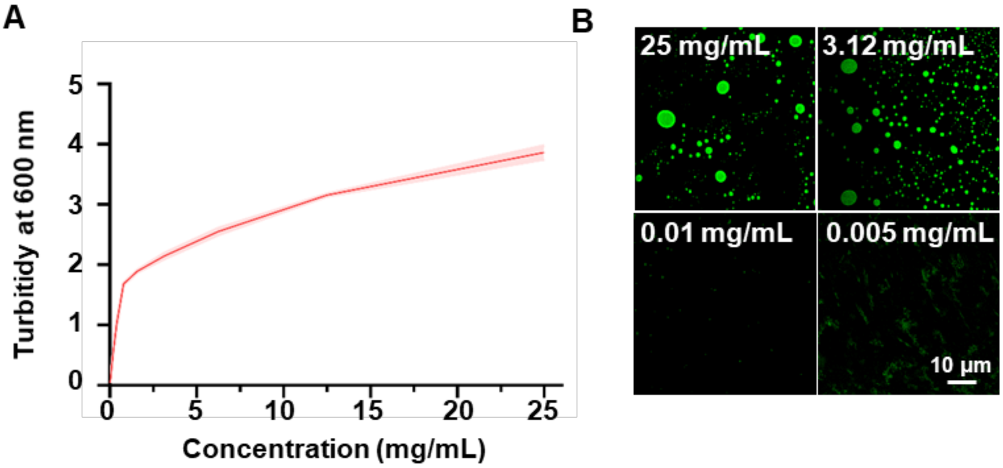

Figure S2. Characterization of curcumin coacervates. (**A**) Turbidity of the solution of curcumin coacervates at different concentrations in a solvent of DMSO: H_2O at a ratio of 1:10. Data are presented as mean ± standard deviation (n=3 independent samples). (**B**) Fluorescent microscopy images of curcumin droplets in different concentrations. The excitation and emission wavelengths of the fluorescent images were set at 488 nm and 525 nm, respectively.

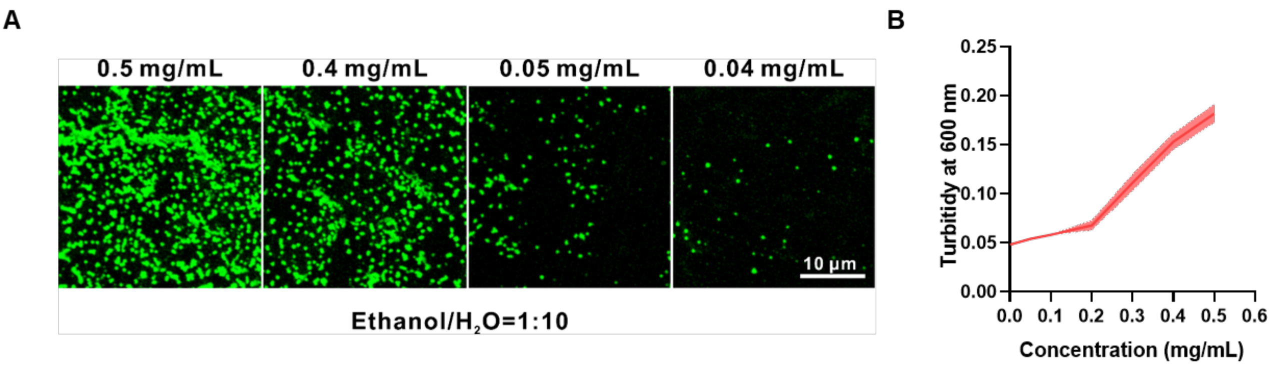

Figure S3. Curcumin coacervates derived from a different organic solvent, ethanol. (**A**) Fluorescent microscopic images of the coacervate droplets formed by curcumin pre-dissolved in ethanol, then diluted to a solvent of ethanol: H_2O at a ratio of 1:10. (**B**) Turbidity of curcumin coacervates at different concentrations in ethanol/water.

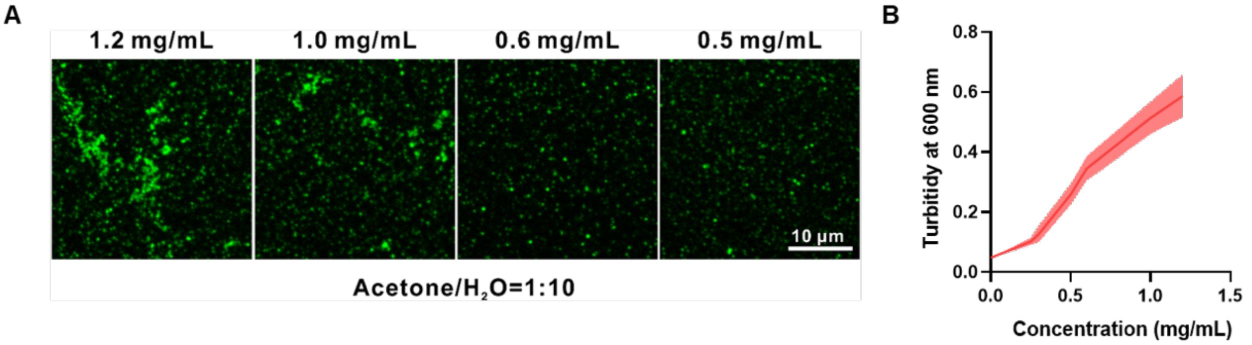

Figure S4. Curcumin coacervates derived from a different organic solvent, acetone. (**A**) Fluorescent microscopic images of the coacervate droplets formed by curcumin pre-dissolved in acetone, then diluted to a solvent of acetone: H_2O at a ratio of 1:10. (**B**) Turbidity of curcumin coacervates at different concentrations in acetone/water.

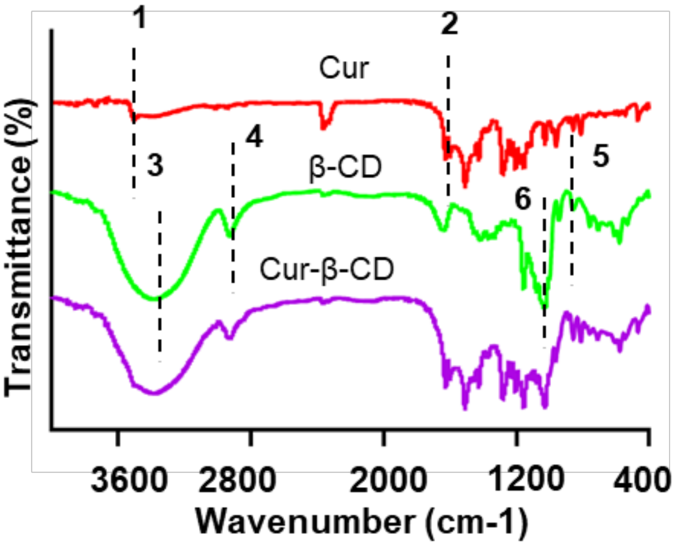

Figure S5. UV-Vis and IR spectra of curcumin-β-CD supramolecular complexes. Briefly, the complex had 1: phenolic OH stretching vibration; 2: stretch vibrations of the benzene ring, and C=O and C=C vibrations of curcumin, and 3-4: the vibrations of the O-H and C-H stretching vibrations; 5: C-O-C of rings; and 6: C-O-C glucose units of β-CD.

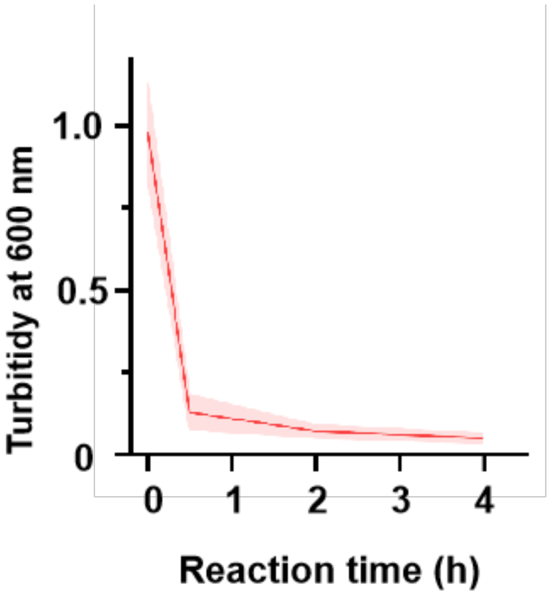

Figure S6. Turbidity of curcumin coacervates after the addition of β-CD. Curcumin, 1 mg/mL; β-CD, 5 mg/mL.

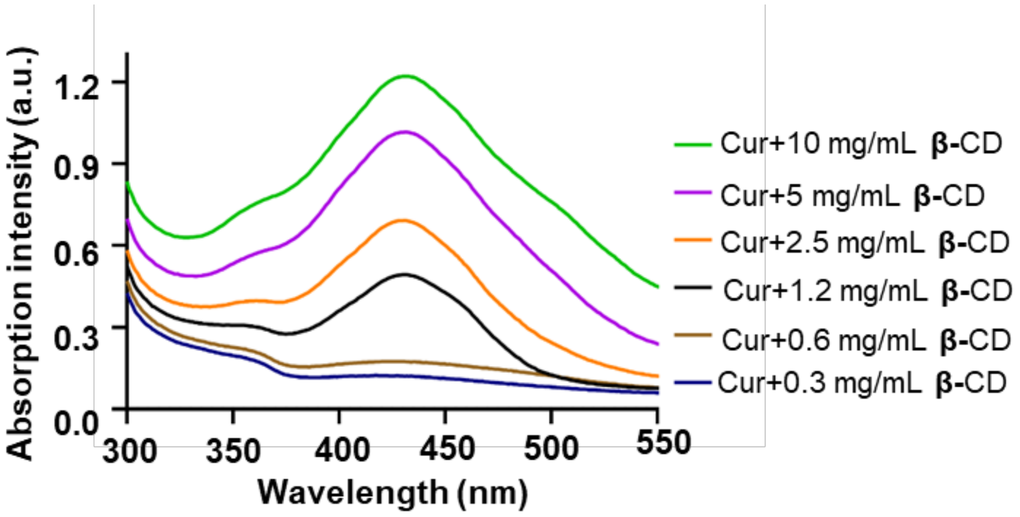

Figure S7. UV-Vis spectra of curcumin-β-CD supramolecular complexes at different concentrations of β-CD. Briefly, 1 mg/mL curcumin coacervate droplets were prepared, and different concentrations of β-CD were added, thoroughly mixed for 0.5 h, and then centrifuged to collect the supernatant for absorption spectroscopy.

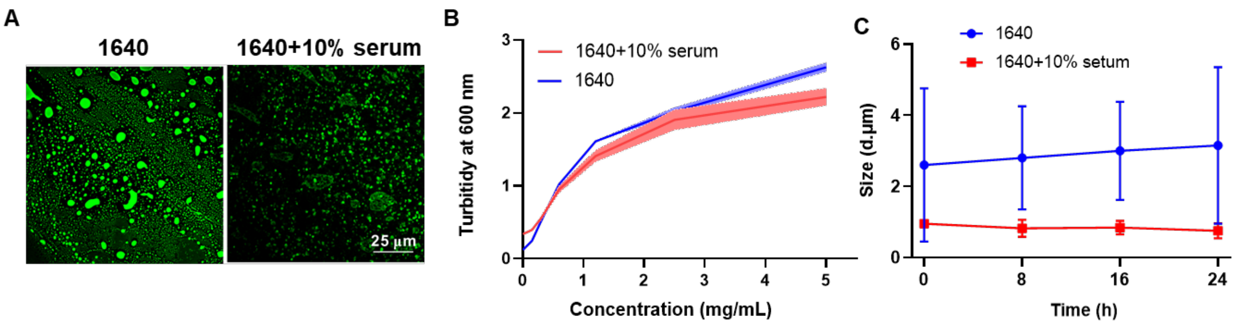

Figure S8. Curcumin coacervates in cell culture medium. (**A**) Fluorescent images, (**B**) turbidity, and (**C**) particle size of curcumin coacervates in RPMI 1640 cell culture medium with or without 10% serum..

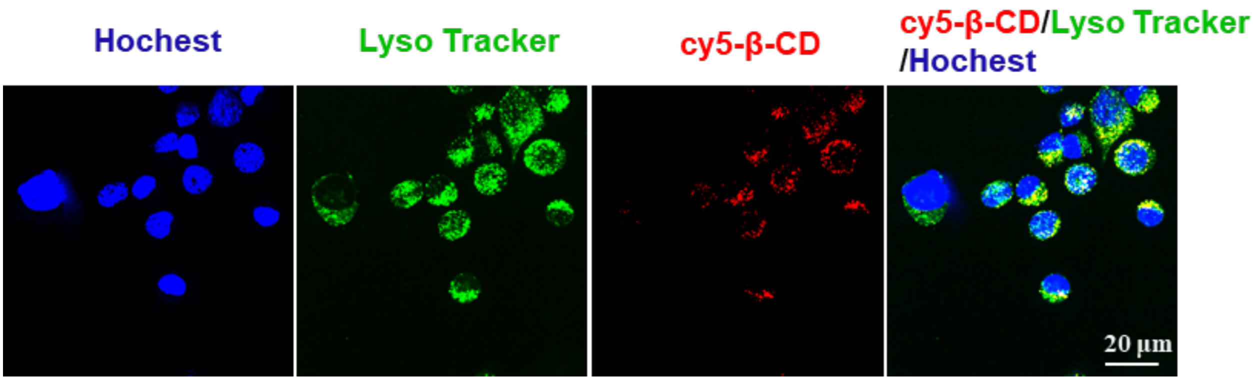

Figure S9. Fluorescent microscopy images of Cy5-β-CD co-stained with lysosomes. Cy5-β-CD (100 μg/mL) was incubated with CT26 cells, along with Lysotracker, for 2 h. The excitation and emission wavelengths were set at 652 nm and 670 nm for the red channel, 488 nm and 525 nm for the green channel, and 405 nm and 460 nm for the blue channel, respectively.

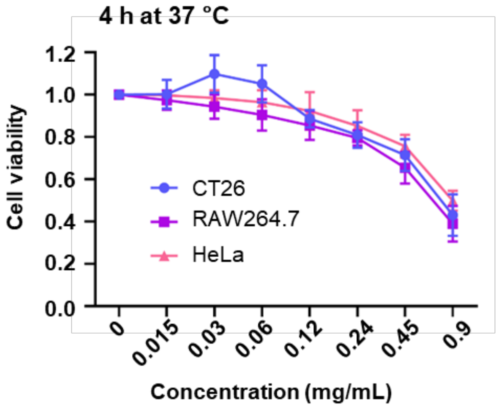

Figure S10. Cytotoxicity of curcumin coacervates in CT26, RAW264.7, and HeLa cells measured based on the CCK-8 assay. Different concentrations of curcumin coacervate droplets were mixed with cells for 4 h before measurements were taken.

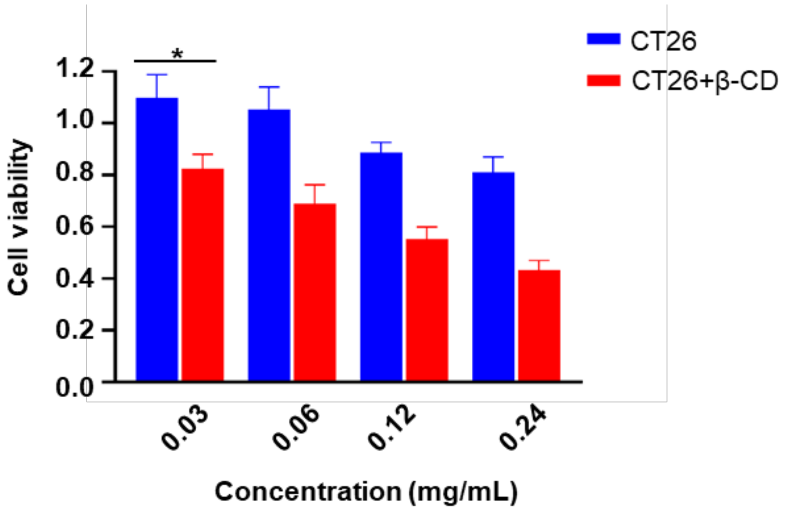

Figure S11. Cytotoxicity of curcumin coacervates in CT26, RAW264.7, and HeLa cells measured based on the CCK-8 assay before and after the addition of β-CD. β-CD, 2 mg/mL. *: $p<0.05$.

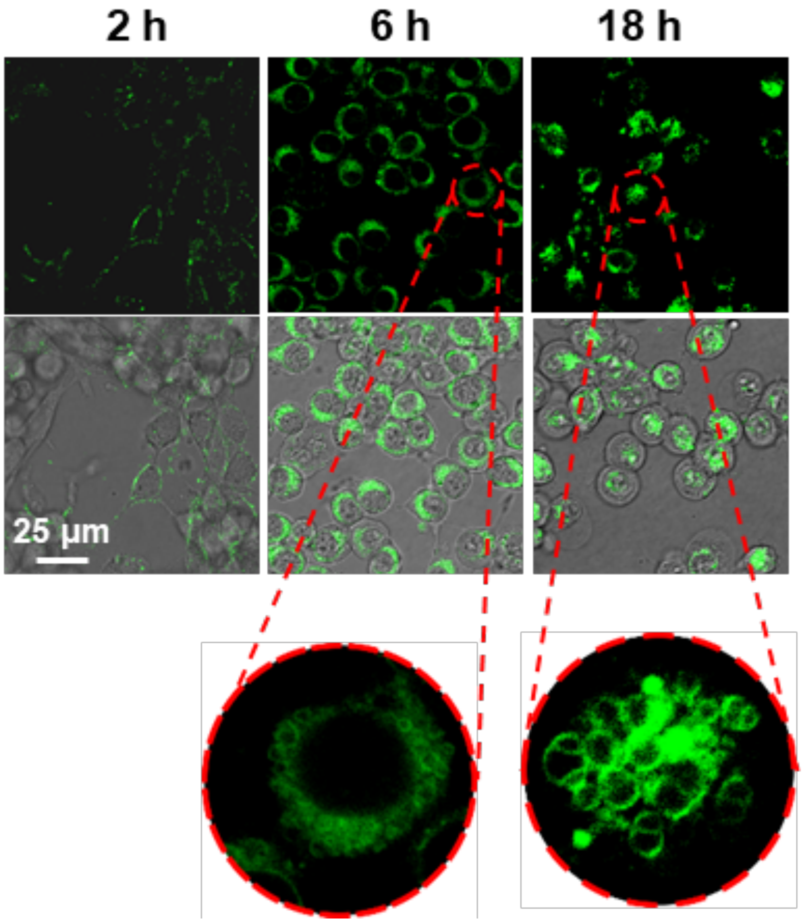

Figure S12. Fluorescent microscopy images of CT26 cells after incubation with the curcumin coacervates. The excitation and emission wavelengths of the fluorescent images were set at 488 nm and 525 nm, respectively. Curcumin coacervates, 100 µg/mL.

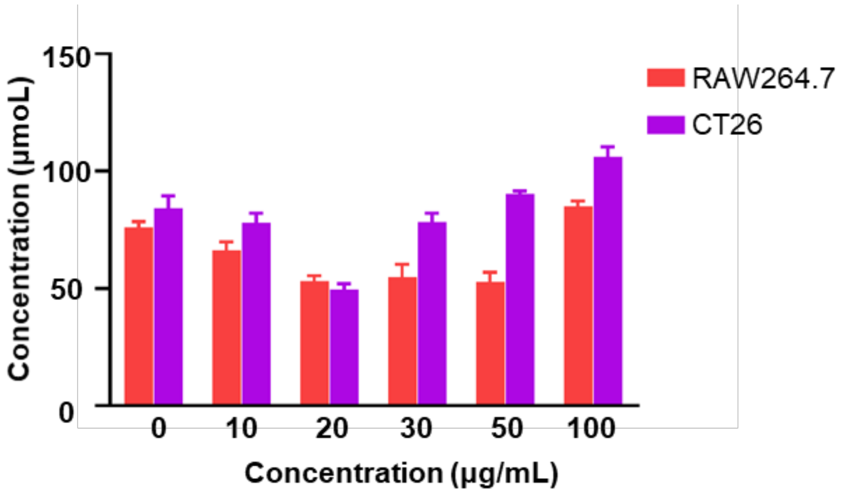

Figure S13. Caspase 4 activity measurement based on the colorogenic reaction of a caspase 4 substrate of curcumin-coacervate-treated cells.

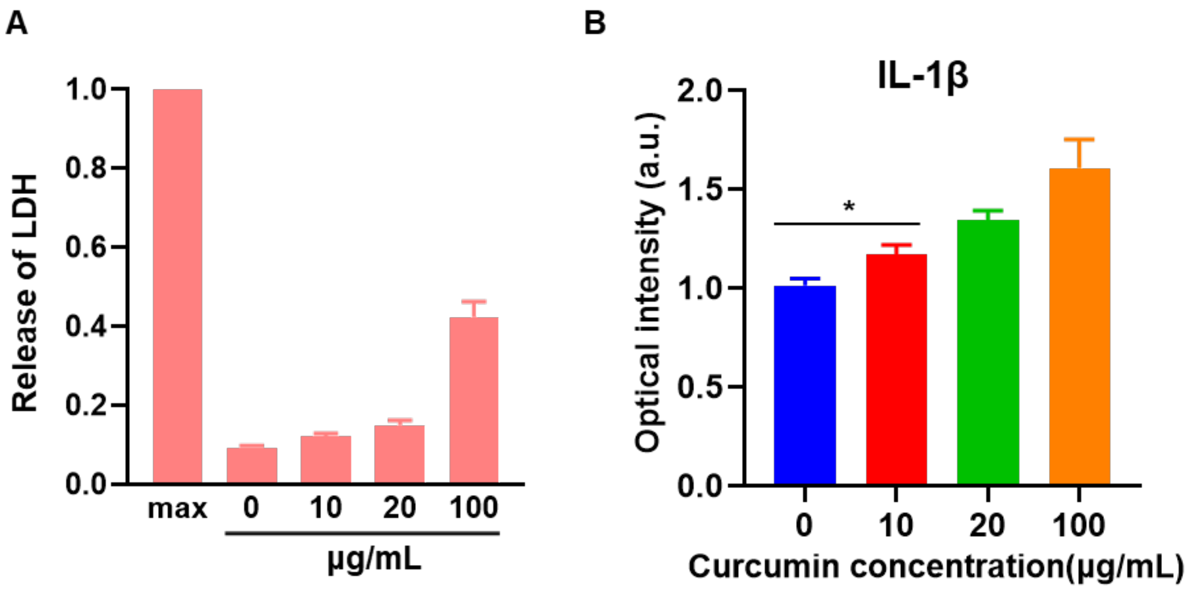

Figure S14. Measurement of LDH and IL-1b from curcumin-coacervate-treated cells.

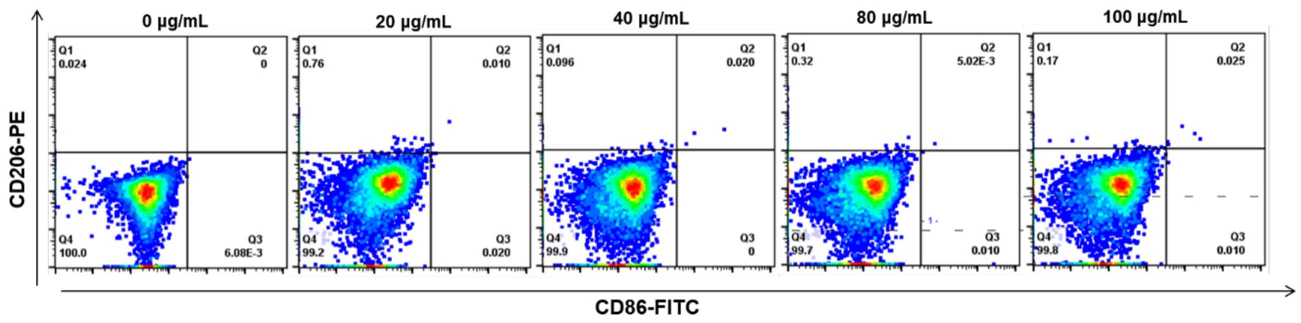

Figure S15. Flow cytometry data of curcumin-coacervate-treated RAW264.7 macrophages. Cells were treated for 32 hours before the measurement.

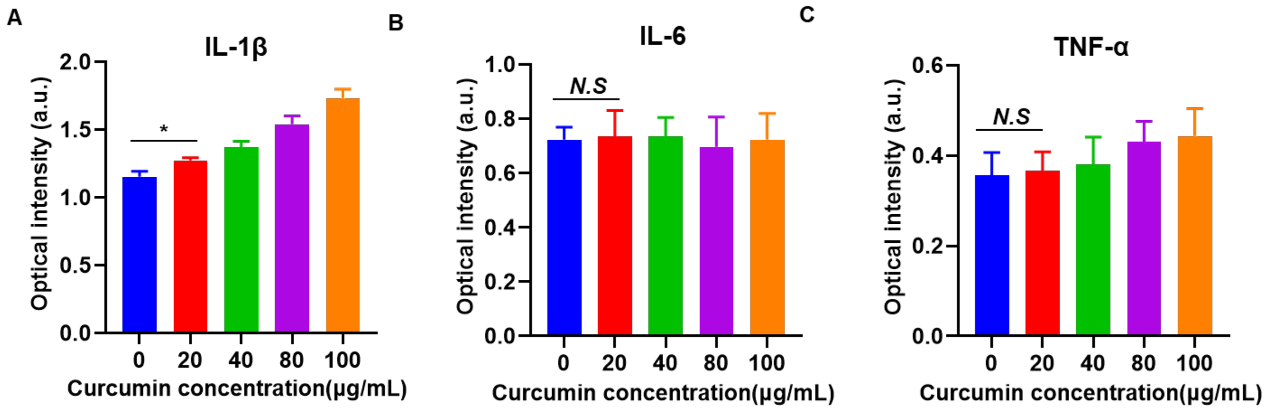

Figure S16. ELISA-based measurement of the secretion of IL-1β, IL-6, and TNF-α from curcumin-coacervate-treated RAW264.7 macrophages.

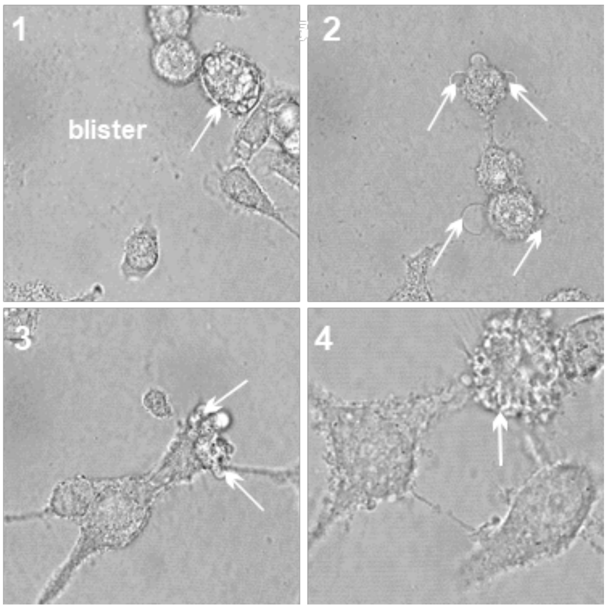

Figure S17. Morphology changes of curcumin-coacervate-treated CT26 cells.

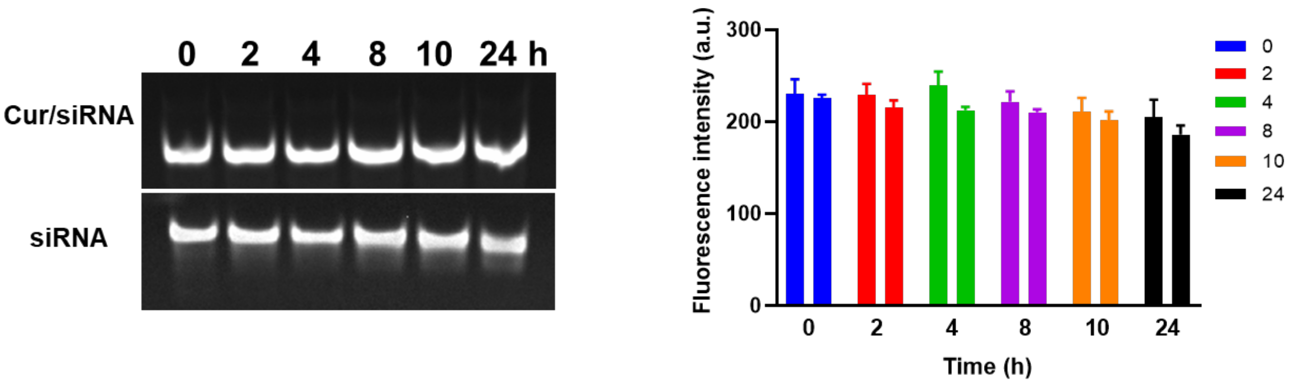

Figure S18. Stability of double-stranded siRNA with or without coacervates in cell culture medium. 12.5% agarose gel electrophoresis of siRNA-loaded coacervates and siRNA after incubation with 1640+10% serum was performed. The siRNA bands were quantified.

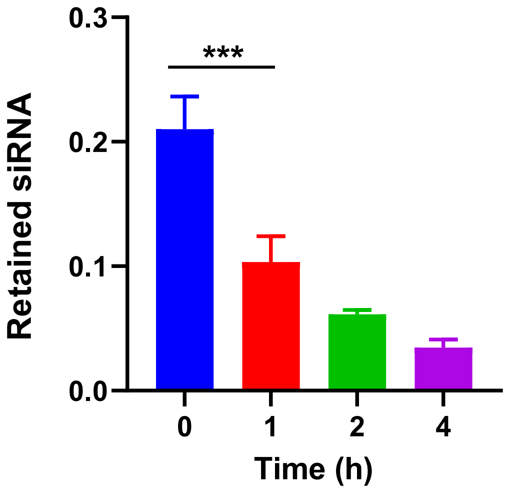

Figure S19. β-CD promotes the release of siRNA from curcumin coacervates.

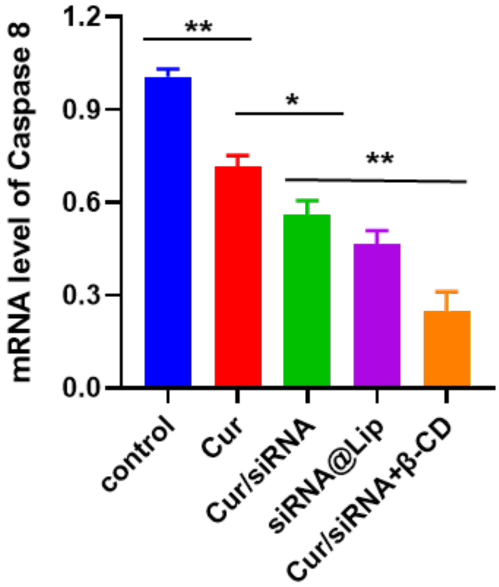

Figure S20. mRNA level of Caspase 8 in CT26 cells after 24-hour treatment with different formulations.

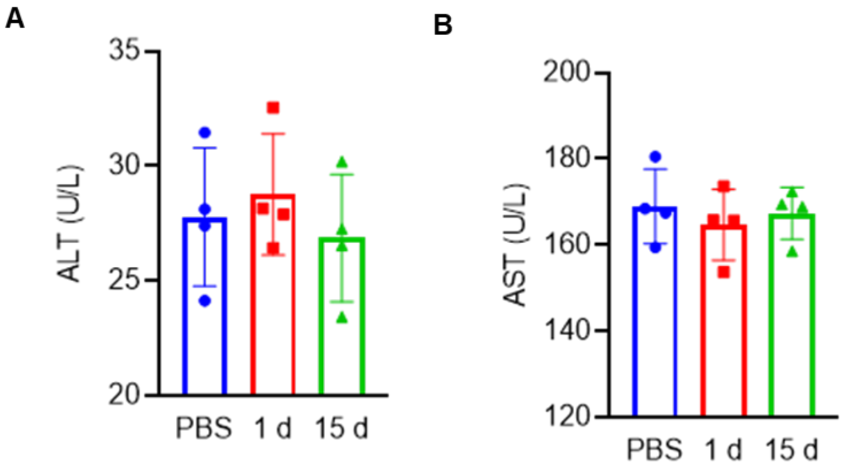

Figure S21. Quantification of the liver function indicators ALT and AST.

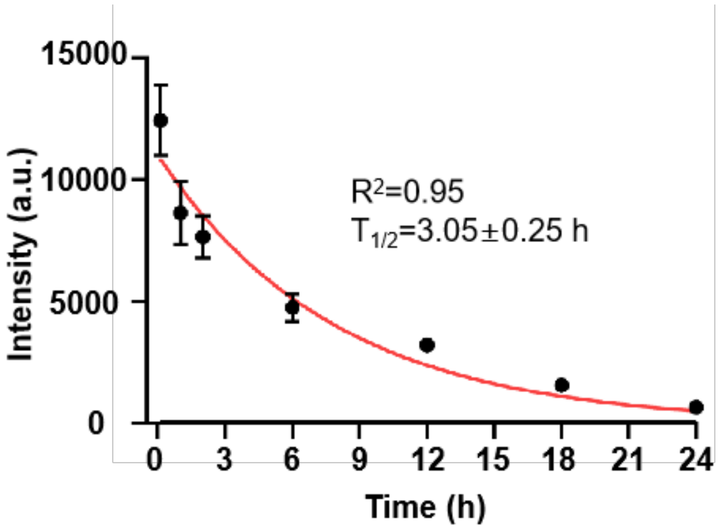

Figure S22. Measurement of the circulating curcumin coacervates in the blood.

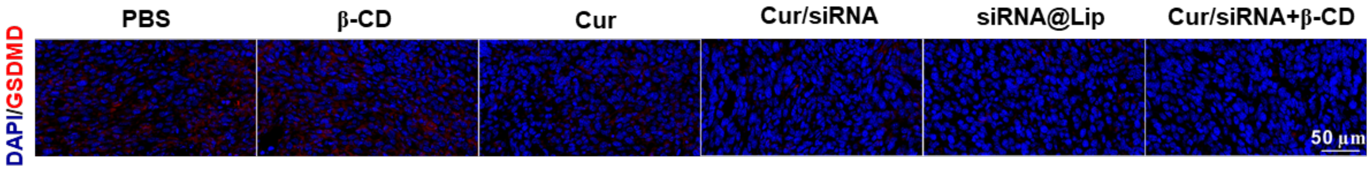

Figure S23. Tumor tissue slides stained for GSDMD showing signs of pyroptosis.

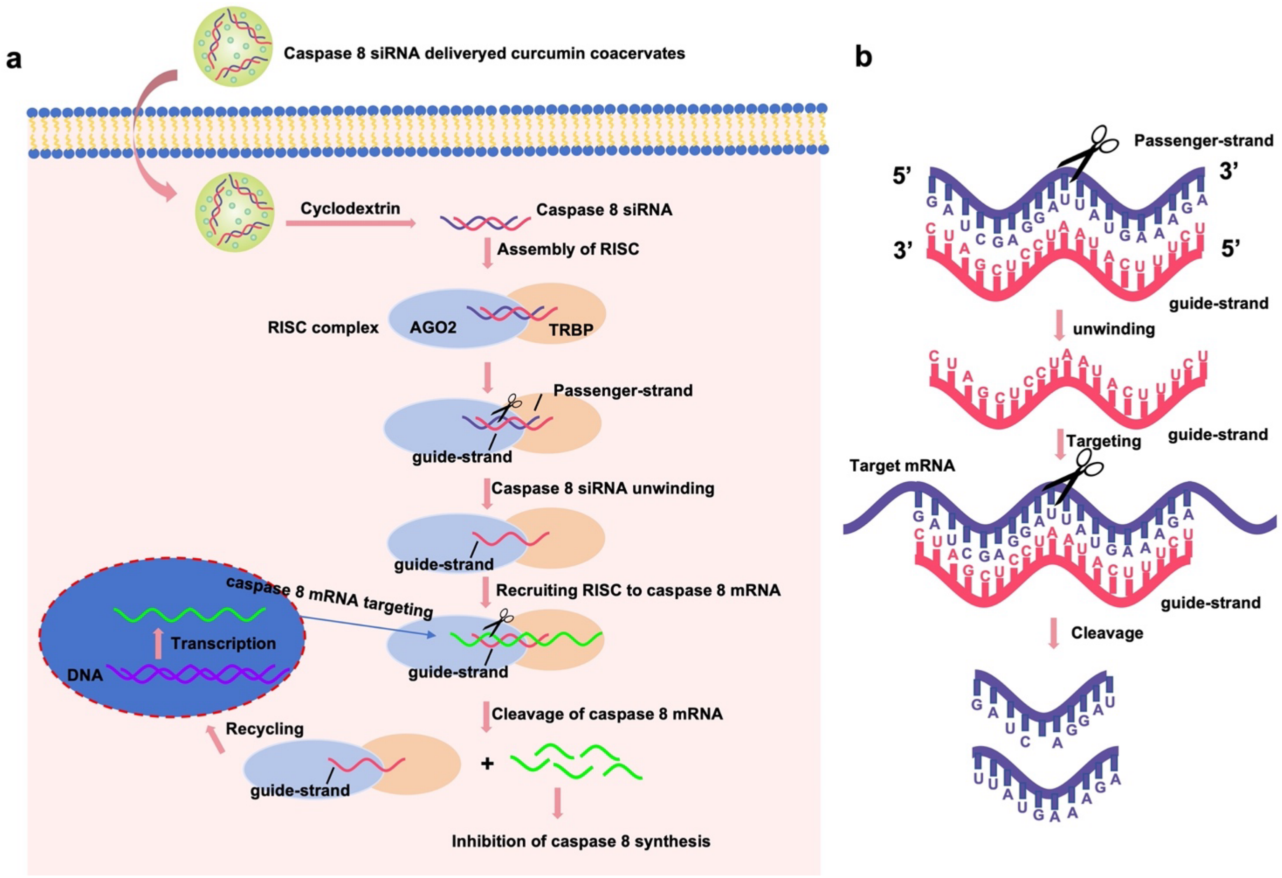

Scheme S1. Schematic illustration showing the mechanism of action of caspase 8 siRNA for cancer treatment. (**A**) Schematic diagram of the proposed mechanism of the Caspase 8 siRNA. Caspase 8 siRNA takes effect through the following steps. 1. Caspase 8 siRNA binds to the RNA-induced silencing complex (RISC) with the Argonaute protein AGO2 as the core component, and the ATP-dependent helicase unwinds the duplex, retaining the guide strand (red) and degrading the passenger strand (blue). 2. The RISC-guide strand complex recognizes the complementary sequence of Caspase 8 mRNA by base pairing, and the PIWI domain of AGO2 cleaves between base 10 and base 11 of the siRNA on the target mRNA. 3. The cleaved mRNA fragment is rapidly degraded by ribonucleases (e.g., XRN1) without being translated into the caspase 8 protein, leading to the down-regulation of caspase 8. (**B**) Sequence information of caspase 8 siRNA. Guide-strand: UCUUUCAUAAUCCUCGAUC, passenger-strand: GAUCGAGGAUUAUGAAAGA.

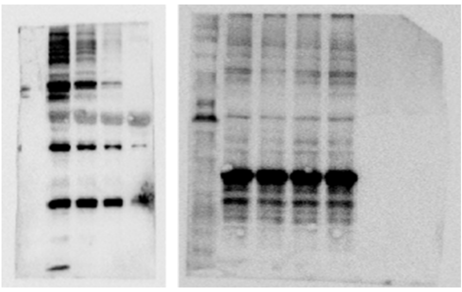

The uncropped gel image of **Figure 4C**.

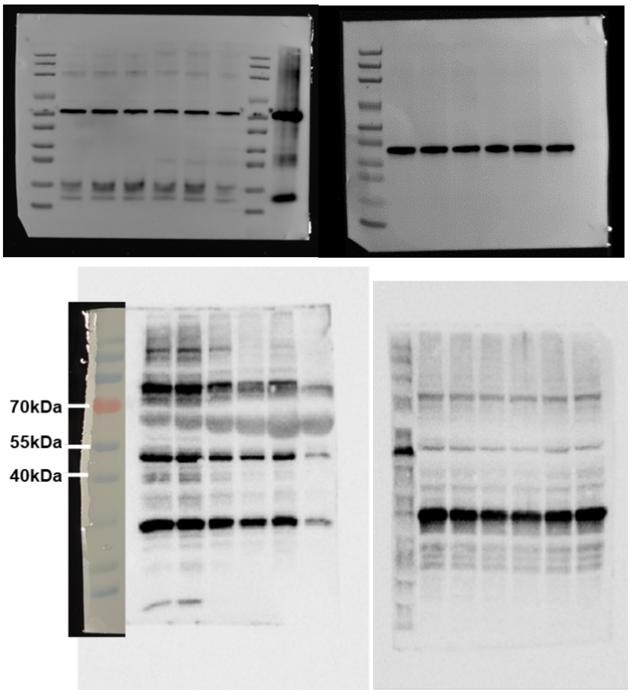

The uncropped gel image of **Figure 5H**.
